# Macrocycle-based covalent organic networks for ultrafast sub–1-Å precision ion sieving

**DOI:** 10.1126/sciadv.aed0804

**Published:** 2026-04-17

**Authors:** Xiao-Gang Jin, Fengrui Yang, Hoei Ying Lim, Rui Jia, Xiao-Hua Ma, Zhe Yang, Chuyang Y. Tang

**Affiliations:** ^1^ School of Chemical Engineering, East China University of Science and Technology, Shanghai 200237, China.; ^2^ UQ Dow Centre for Sustainable Engineering Innovation, School of Chemical Engineering, The University of Queensland, St. Lucia, QLD 4072, Australia.; ^3^ Department of Civil and Environmental Engineering, The Hong Kong University of Science and Technology, Clear Water Bay, Kowloon, Hong Kong SAR 999077, China.; ^4^ Department of Civil Engineering, The University of Hong Kong, Pokfulam, Hong Kong SAR 999077, P.R. China.

## Abstract

The water-energy nexus demands membranes with subångström ion-sieving precision, a capability hindered by the intrinsic selectivity limits of conventional materials. We fabricated ultrathin covalent organic network (CON) membranes via interfacial polymerization of 1,4,7,10-tetraazacyclododecane (cyclen) with either terephthaloyl chloride or isophthaloyl chloride. The resulting architectures demonstrate narrow pore size distribution and subångström-level ion resolution. CON-T membranes exhibit exceptional water permeance (22.2 liters per square meter per hour per bar) along with Li^+^/Mg^2+^ and Cl^−^/SO_4_^2−^ selectivities of 82.6 and 118.1, respectively. Monomer conformation engineering yielded CON-I membranes that set high benchmarks for Li^+^/Mg^2+^ (326.7) and Cl^−^/SO_4_^2−^ (376.9) selectivity. At the process scale, CON-T membranes facilitate highly efficient lithium resource extraction (97.4% recovery with 99.3% purity), while CON-I membranes enable Cl^−^ production at ~99.9% purity from industrial wastewater. This methodology establishes a paradigm for high-resolution ion-sieving membranes that is fully compatible with industrial-scale polymeric membrane manufacturing, offering a viable solution for next-generation water-energy systems.

## Teaser

Covalent organic network membranes with subångström-level ion resolution enable ultrafast precise sieving of mono/divalent ions.

## INTRODUCTION

Achieving high permeability and selective ion transport across membranes is crucial in applications such as nanofluids, ion separation, and energy conversion (*1*–*5*). For example, the efficient separation of Li^+^ and Mg^2+^ helps to extract lithium resources from salt lakes, improving the efficiency and economics of lithium recovery to meet the growing demand for lithium (*6*–*8*), while the efficient separation of Cl^−^ and SO_4_^2−^ is of great significance for treating coal chemical wastewater, enabling salt recovery and zero liquid discharge (*9*, *10*). Precise separation of ions is one of the main goals of membrane separation, and the central challenge is to design membrane materials with subnanometer precise pore structures and controlled interfacial chemistry to distinguish ions of similar size but different valence (*5*, *11*).

Although membrane technology has been widely used in water treatment, desalination, drinking water purification, and wastewater management (*12*, *13*), thin-film composite membranes made via interfacial polymerization (IP) reaction still face a fundamental permeance-selectivity trade-off (*14*, *15*). The selective layer of the membrane is typically formed by a cross-linked polyamide network, produced through a rapid IP reaction between amine and acyl chloride monomers (*16*). However, the kinetics of this reaction is difficult to control, resulting in the formation of polymer networks with wide pore size distributions and high chemical heterogeneity, which severely limits their ability to accurately screen mono/divalent ions (*17*–*20*).

Emerging materials such as covalent organic frameworks and two-dimensional porous polymers have been seen as potential approaches to break the above trade-off effect because of their designable pore structure and surface chemistry (*21*–*23*). However, these materials often rely on complex solvothermal synthesis or mechanical stripping processes (*24*, *25*), which are difficult to be compatible with existing large-scale preparation processes for rolled membrane modules (*26*–*28*). Therefore, it remains challenging to achieve precise subnanometer-scale tuning of membrane pore structure and chemistry while preserving the scalability of IP techniques.

A covalent organic network (CON) is a smart material that combines the topological ordering of covalent organic frameworks with the scalable manufacturability of amorphous polymer membranes (*29*, *30*). Their structural versatility and exceptional solvent stability have positioned them as leading candidates for high-precision separation applications (*31*–*33*). By leveraging macrocyclic amine monomers that form robust covalent bonds with dichloride, CON can achieve an ordered and highly interconnected microporous architecture (*34*, *35*). This unique structural design has inspired and prompted us to develop the advanced ion-selective membranes.

Herein, ultrathin CON membranes were successfully prepared by IP reaction using 1,4,7,10-tetraazacyclododecane (cyclen) and terephthaloyl chloride (TPC) or isophthaloyl chloride (IPC) as the building blocks. The pore size of the CON membranes can be adjusted by changing the positions of the chloride groups (meso- and para-sites). A combination of experiments and molecular dynamics (MD) simulations verified the ability and potential mechanism of ultrafast mono/divalent ion sieving of CON membranes. In addition to coupon-scale performance testing, we further analyzed membrane performances at process-scale analysis, which confirms the industrial application potential of CON membranes. Thus, this work provides an elegant strategy for the preparation of high-performance ionic precision sieving membranes using a well-established IP process.

## RESULTS

### Preparation and physicochemical properties of CON membranes

**Fig. 1.**
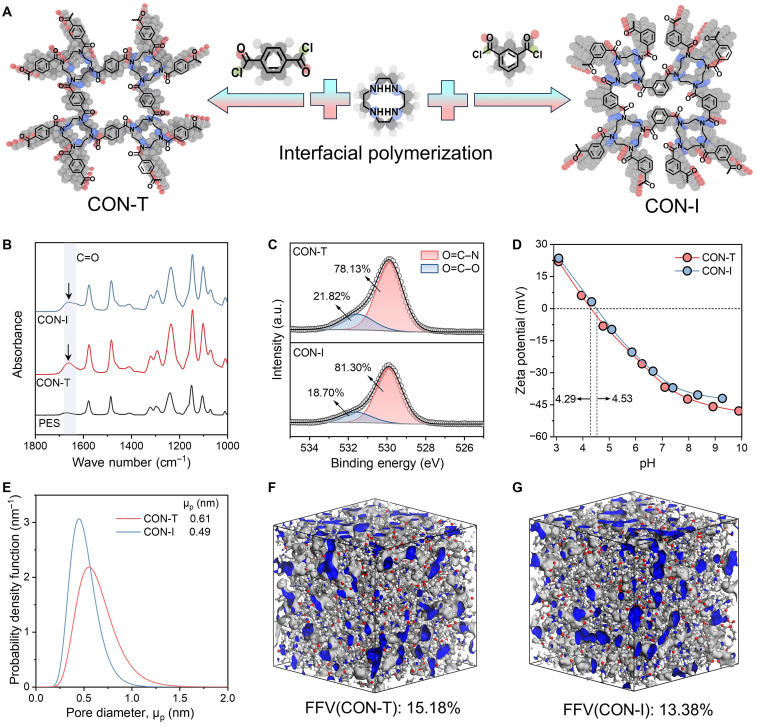
Preparation and physicochemical properties of CON membranes. (**A**) Cyclen and TPC (left) or IPC (right) as building blocks for the formation of CON structures. At the water/oil reaction interface, the acyl chloride group and amino group undergo a condensation reaction to form an amide bond. (**B**) FT-IR spectra of the PES substrate (black) and CON-T (red) and CON-I (blue) membranes. (**C**) High-resolution spectra of deconvoluted O 1s of CON-T (top) and CON-I (bottom) membranes. a.u., arbitrary units. (**D**) Zeta potential of CON-T (red) and CON-I (blue) membranes at different pH levels (from 3 to 10). The dotted line is used to indicate the isoelectric point (pH value when the zeta potential is equal to 0) of the membranes. (**E**) Pore size distribution of CON-T (red) and CON-I (bule) membranes obtained by neutral solute filtration experiment; see text S1and fig. S2C for detailed information. (**F** and **G**) Snapshot and fractional free volumes (FFVs) of CON-T (F) and CON-I (G) membrane structures by MD simulation. Gray indicates an occupied space by polymer skeleton, and blue indicates free volume. The radius of the probe is 1 Å. Details for MD simulation methods can be found in text S2.

The condensation reaction between amino groups and acyl chlorides has contributed to the classic formation of a polyamide rejection layer of reverse osmosis and nanofiltration membranes in water treatment. Similarly, the condensation reaction of cyclen with diacyl chloride can produce a polymer network with a high degree of connectivity (Fig. 1A). The structure of this network can be tailored according to the conformation of the diacyl chloride monomer (for example, symmetric for TPC and asymmetric for IPC). Such a reaction produced continuous defect-free and robust ultrathin free-standing nanofilms (fig. S1, A and B). Considering the mechanical strength required for pressure-driven membrane separation processes, the CON membranes were prepared by a well-integrated in situ IP process on a commercial polyethersulfone (PES) substrate to form a thin-film composite structure (fig. S1, C and D). CON membranes prepared using TPC and IPC as organic phase monomers under optimal conditions were labeled as CON-T and CON-I membranes, respectively. Detailed information on preparation conditions can be found in the “Fabrication of CON membranes” section.

Figure 1B shows that a new strong peak was detected at 1660 cm^−1^ for CON-T and CON-I membranes in Fourier transform infrared spectroscopy (FT-IR) spectra, indicating that the ─NH of cyclen cross-linked with the acyl chloride group of TPC or IPC to generate amide bonds (*36*). In addition, the N 1s peak on the x-ray photoelectron spectrum strongly validates the FT-IR results (fig. S2A and table S1). Notably, by comparing the deconvoluted O 1s, the N─C═O content in the CON-I membrane is higher than that in the CON-T membrane, while the O─C═O content is relatively low (Fig. 1C). Correspondingly, CON-T membranes have a lower isoelectric point (Fig. 1D) and stronger hydrophilicity (fig. S2B), which results from the hydrolysis of residual acyl chloride groups into carboxylic groups.

Neutral solute filtration experiments and MD simulations were used for in-depth analysis of CON-T and CON-I pore structures. The molecular weight cutoff of CON-T was 376 Da with an average diameter of 0.61 nm, while the molecular weight cutoff of the CON-I membrane was 234 Da with an average diameter of 0.49 nm and a much narrower pore size distribution (fig. S2C and Fig. 1E). MD simulations constructed membrane models containing interconnected subnanoscale voids, showing pore diameter distributions primarily between 4 and 7 Å (fig. S2D). In addition, the CON-T membrane (15.18%) shows a higher fractional free volume (FFV) compared to the CON-I membrane (13.38%) (Fig. 1, F and G). Collectively, these findings indicate that both membranes have well-defined subnanopore structures, while the CON-I membrane has a smaller and more uniform membrane pore size.

### Morphology of CON membranes

**Fig. 2.**
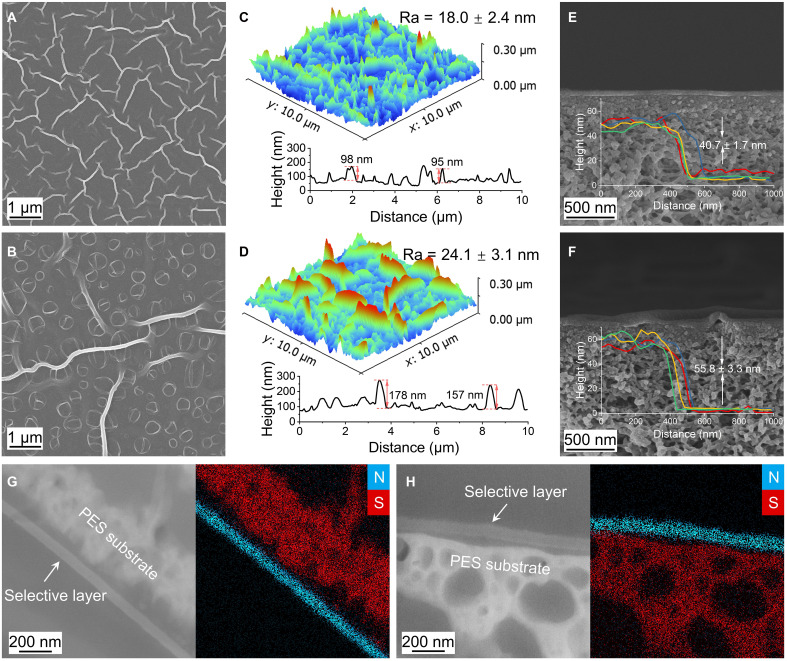
Morphological of CON membranes. (**A** to **D**) Surface morphology of CON-T [(A) and (C)] and CON-I [(B) and (D)] membranes by SEM [(A) and (B)] and AFM [(C) and (D)]. The line graph presents height variations across the three-dimensional surface morphology of the membrane, including features such as stripes and coffee-ring structures. (**E** and **F**) Cross-sectional morphology of CON-T (E) and CON-I (F) membranes by SEM. The inset shows the thickness of the selective layer tested with AFM. (**G** and **H**) Dark-field STEM cross-sectional images and corresponding STEM-EDX elemental mappings of CON-T (G) and CON-I (H) membranes, where “N” (blue) denotes nitrogen, and “S” (red) denotes sulfur.

The conformation of the acyl chloride monomer is also reflected in the morphological features of the CON membrane. According to field emission scanning electron microscopy (SEM), the CON-T membrane exhibits a striped structure, while a special morphology with the coexistence of coffee-ring and striped structures was observed on the surface of the CON-I membrane (Fig. 2, A and B). On the basis of three-dimensional imaging of membrane morphology using atomic force microscopy (AFM), the average surface roughness (Ra) values of CON-T and CON-I were 18.0 ± 2.4 and 21.4 ± 3.1 nm, respectively (Fig. 2, C and D). We note that the stripe height of the CON-I membrane is higher than that of the CON-T membrane. Moreover, CON-T exhibited a higher surface area ratio (6.56 ± 0.86%) than CON-I (3.39 ± 0.12%), indicating a larger effective filtration area. The acyl chloride groups in TPC are located in the para position of the benzene ring, and the strong polarity of the acyl chloride groups is concentrated on both sides of the benzene ring to form a symmetric dipole distribution. In IPC, the acyl chloride groups are located in the meso position of the benzene ring, and the polar groups are skewed to one side, with the dipole moments oriented away from the symmetry axis. The difference in symmetry and polarity distribution of the molecules will lead to different orientations at the water/oil interface, resulting in different morphological characteristics after the IP process. In addition, another important factor could be the different diffusion rate of the aqueous-phase monomer (*16*). Considering the macrocyclic nature of the aqueous-phase monomer (i.e., cyclen), the two different acyl chloride monomers (i.e., TPC and IPC) might exhibit substantially different steric hindrance during the IP reaction, thereby affecting the corresponding diffusion rate of cyclen. Similar types of three-dimensional structures have been reported to notably increase the effective mass transfer area of the membrane as well as optimize transport pathways (*16*, *37*–*39*), thereby improving membrane permeability.

The ultrathin and dense selective layer can be observed from the SEM cross-sectional image (Fig. 2, E and F). The thickness of the CON selective layers of the membranes was further analyzed by AFM, which was 40.7 ± 1.7 nm for the CON-T membrane and 55.8 ± 3.3 nm for the CON-I membrane. Furthermore, scanning transmission electron microscopy-energy-dispersive x-ray spectroscopy (STEM-EDX) elemental mapping reveals the interface between the CON selective layer (nitrogen-rich) and the PES substrate (sulfur-rich) (Fig. 2, G and H). Furthermore, the ridge structures visible in the low-magnification TEM images (fig. S3) are consistent with the three-dimensional topology evidenced in the corresponding SEM and AFM images. Overall, the CON-T membrane is more structurally organized and thinner, demonstrating superior permeability potentials.

### Separation performance of CON membranes

**Fig. 3.**
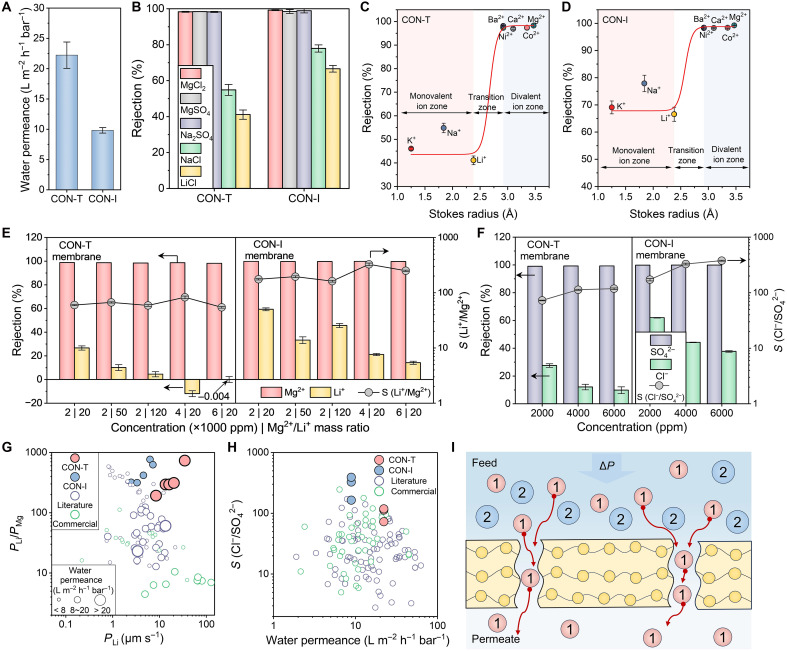
Separation performance of CON membranes. (**A** and **B**) Water permeance (A) and salt rejection (B) of CON-T and CON-I membranes. L, liter; h, hour. (**C** and **D**) Rejection of different solutes as a function of Stokes radius by CON-T (C) and CON-I (D) membranes. The monovalent ion region is denoted by light red, while the divalent ion region is represented by light blue. Detailed information can be referred to table S4. In (B) to (D), the feed concentration was 1000 ppm. (**E**) Ion (Mg^2+^ and Li^+^) rejection and Li^+^/Mg^2+^ selectivity [*S* (Li^+^/Mg^2+^)] of CON-T and CON-I membranes. The feed composition is defined by the total salt concentration (ppm) and Mg^2+^/Li^+^ mass ratio. (**F**) Ion (SO_4_^2−^ and Cl^−^) rejection and Cl^−^/SO_4_^2−^ selectivity [*S* (Cl^−^/SO_4_^2−^)] of CON-T and CON-I membranes. The feed concentration ranges from 2000 and 4000 to 6000 ppm, while the mass concentration ratio of NaCl to Na_2_SO_4_ is maintained at 1:1. In (A) to (F), the test pressure was 6.0 bar, the temperature was 25 ± 1°C, the cross-flow velocity was 22.4 cm s^−1^, and the effective membrane area was 22.05 cm^2^. The error bars represent the standard deviation of data from at least three distinct samples. (**G**) Comparison of Li^+^/Mg^2+^ separation performance of state-of-the-art nanofiltration membranes reported in the literature and commercial, CON-T, and CON-I membranes. The symbol size reflects the water permeance (liter m^−2^ hour^−1^ bar^−1^) of the membranes. Detailed data can be found in table S5. (**H**) Comparison of the Cl^−^/SO_4_^2−^ separation performance of state-of-the-art nanofiltration membranes reported in the literature and commercial, CON-T, and CON-I membranes. Detailed data can be found in table S6. (**I**) The schematic diagram illustrates the separation of mono/divalent ions by a CON membrane, where monovalent and divalent ions are denoted by symbols 1 and 2, respectively, regardless of specific ion charge.

To optimize membrane separation performances, the effects of cyclen concentration, reaction time, and sodium lauryl sulfate (SLS) concentration on CON membrane permeation selectivity were systematically investigated (figs. S4 to S6). Subsequent investigations examined the influence of substrates on CON membrane formation (tables S2 and S3) (*40*, *41*), and the membranes prepared using a conventional monomer also served as the comparison benchmark (figs. S7 and S8). Under optimal conditions, CON-T membranes demonstrate an excellent water permeance of 22.2 liters m^−2^ hour^−1^ bar^−1^, which is notably higher than that of CON-I membranes (9.8 liters m^−2^ hour^−1^ bar^−1^). This enhanced water permeance of CON-T membrane can be attributed to the lower cross-linking degree (resulting in larger pore size), higher free volume, thinner selective layer thickness, larger effective filtration area, and greater hydrophilicity of the former. In terms of selectivity, the rejection of divalent ions, i.e., Na_2_SO_4_, MgSO_4_, and MgCl_2_, by both membranes remained above 98%. In addition, the denser structure of CON-I membranes resulted in higher rejection of NaCl and LiCl (Fig. 3, A and B). Furthermore, both CON-T and CON-I membranes exhibit operational stability, maintaining nearly constant water flux and salt rejection over a continuous operation of 216 hours (fig. S9, A and B). In addition, both types of membranes show good stability and a linear relationship between water flux and applied pressure over a wide range of operating pressures (from 2.0 to 10.0 bar) (fig. S9, C and D).

To assess membrane ion-ion separation, inorganic salts with different cation ion types (KCl, NaCl, LiCl, BaCl_2_, NiCl_2_, CaCl_2_, CoCl_2_, and MgCl_2_) were used to evaluate the ion sieving ability of the CON-T (Fig. 3C) and CON-I (Fig. 3D) membranes. Both membranes retained about 98% of the divalent ions. Meanwhile, the rejection of monovalent ions (K^+^, Na^+^, and Li^+^) by the CON-T membrane was around 50%, and the retention of monovalent ions by the CON-I membrane was higher (>60%) (Fig. 3, C and D, and table S4). It is noteworthy that the rejection of Li^+^ with a larger Stokes radius is slightly lower than that of Na^+^ with a smaller radius, which suggests that the CON membranes are favorable for Li^+^ transport. Under the premise of size sieving, transmembrane transport of ions is favored when the binding energy between ions and cyclen is large (*42*). The preferential penetration of Li^+^ may be related to the binding capacity between Li^+^ and cyclen (*43*, *44*).

Precise sieving of mono/divalent ions has emerged as a key research frontier, essential for energy-efficient separation processes such as resource recovery, brine management, and selective ion extraction (*5*). Among them, Li^+^/Mg^2+^ separation is the key to achieving lithium resource extraction from salt lakes, and Cl^−^/SO_4_^2−^ separation is an essential approach for achieving minimal or zero discharge in industrial brine treatment. Considering that the practical ion separation process often involves complex mixtures, we then examined the ion separation performance of CON-T and CON-I membrane pairs in mixed multielectrolytes, rather than limiting the evaluation to single-salt solutions.

To mimic the actual salt-lake lithium extraction process, we focused on the Li^+^/Mg^2+^ separation of CON-T and CON-I membranes at high Mg^2+^/Li^+^ mass ratios (20, 50, and 120) and different total salt concentrations [i.e., 2000, 4000, and 6000 parts per million (ppm), respectively] (Fig. 3E). The separation factors of CON-T and CON-I membranes fluctuated under different feed conditions because of the strong influence of operating and solution conditions on the solute-solute selectivity of the pressure-driven separation membrane process (*5*). Notably, we observed that both CON-T and CON-T membranes exhibited promising Li^+^/Mg^2+^ separation performance under all feed conditions (Fig. 3E). We also observed negative rejection of Li^+^ in the CON-T membrane (Mg^2+^/Li^+^ mass ratio: 20; total salt concentration: 4000 ppm). This phenomenon can be attributed to the restricted rejection of monovalent ions by CON-T membranes. The permeation of Cl^−^ facilitates the permeation of cations with higher permeability (Li^+^) in the system to maintain electroneutrality (*45*, *46*).

We further evaluated the anion selectivity of CON-T and CON-I membranes—specifically for Cl^−^/SO_4_^2−^ separation—using mixed salt solutions containing Na_2_SO_4_ and NaCl. The mass concentrations of Na_2_SO_4_ and NaCl were controlled to be 1:1, and the total salt concentration was varied to investigate the ion sieving ability of CON-T and CON-I membranes in different systems. The separation factors of the CON-T and CON-I membranes gradually increased with the increase in the mixed salt concentration, reaching 118.1 and 376.9, respectively, at a feed concentration of 6000 ppm (Fig. 3F).

It is generally accepted that the selective separation of ions is notably influenced by the charge and ion size (*47*, *48*). The spatial site resistance effect is obvious; the larger the size, the more difficult the transport of ions. On the other hand, the membrane surface charge has the opposite effect on co-ions (with the same charge to the membrane) and counterions (with opposite charge): It impedes the transport of co-ions and promotes that of counterions. Therefore, negatively charged nanofiltration membranes perform well for anion separation (*9*, *49*), while positively charged nanofiltration membranes have been widely investigated for cation separation (*50*, *51*). However, membrane materials capable of accurately sieving both mono/divalent anions and cations are still rare. Our results show that the CON-T and CON-I membranes excel in the simultaneous separation of mono/divalent cations and anions, with permeability and selectivity exceeding that of most commercial nanofiltration membranes and state-of-the-art nanofiltration membranes reported in the literature (Fig. 3, G and H, and fig. S10). The superior permeability of CON membranes is due to the highly connected and ordered pores, and the excellent ionic selectivity is due to the uniformity of their appropriate size distribution. Overall, CON membranes enable high-efficiency separation of mono/divalent ions regardless of ionic charge (Fig. 3I).

### Ion transport mechanism of the CON membrane

**Fig. 4.**
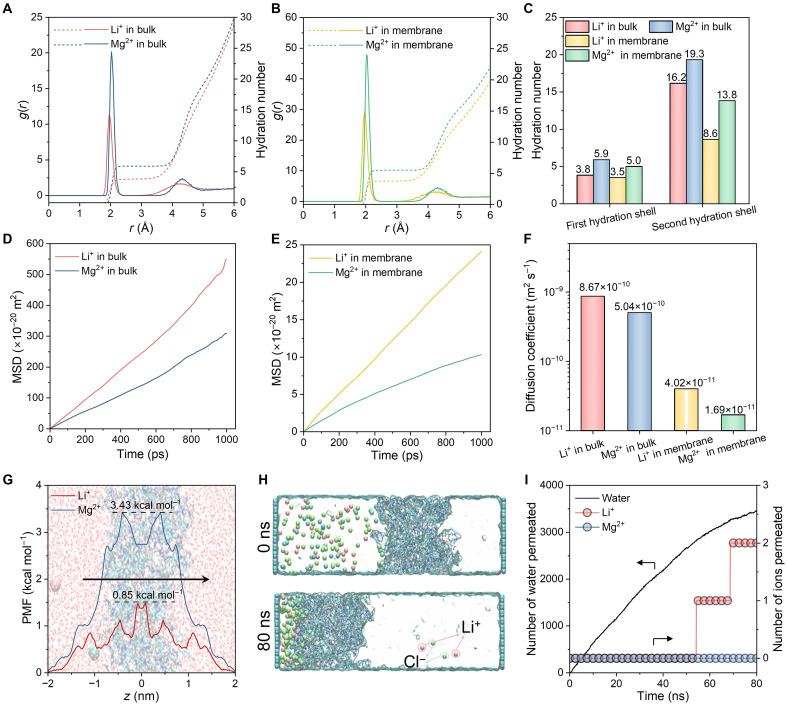
MD simulations reveal the potential mechanism for Li^+^/Mg^2+^ separation. (**A** and **B**) Radial distribution function profile (solid line; see left *y* axis) of ions (Mg^2+^ and Li^+^) and water (O) and hydration number (dotted line; see right *y* axis) in a bulk solution (A) and CON membrane (B). (**C**) Hydration number of ions (Mg^2+^ and Li^+^) in the first and second shells in a bulk solution and CON membrane. (**D** and **E**) Mean square displacement (MSD) profile of ions (Mg^2+^, and Li^+^) in a bulk solution (D) and CON membrane (E). (**F**) Diffusion coefficient of ions (Mg^2+^ and Li^+^) in a bulk solution and CON membrane. (**G**) PMF profile for moving ions (Mg^2+^ and Li^+^) across the CON membrane. (**H**) NEMD simulation snapshots at the initial (0 ns) and final (80 ns) states. The small balls represent ions and are distinguished by color: Pink represents Li^+^, green represents Cl^−^, and blue represents Mg^2+^. (**I**) Number of water molecules (refer to the left *y* axis) and ions (refer to the right *y* axis) passing through the CON membrane in NEMD simulations as a function of simulation time. Please note that the CON membrane used to simulate Li^+^/Mg^2+^ separation was modeling by cyclen and TPC.

To further illustrate ion transport mechanisms for CON membranes, MD simulations were performed to investigate ion trajectories and interactions at the nanoscale. The transmembrane transport of ions involves two primary steps: First, ions are distributed from the bulk phase solution to the CON membrane and then diffuse within the membrane (*52*, *53*). As shown in Fig. 4 (A to C), comparative analysis of radial distribution functions and hydration numbers revealed that Mg^2+^ ions exhibit higher hydration energy than Li^+^ in both a bulk solution and CON membranes, consequently requiring greater energy for dehydration (*54*, *55*), leading to a markedly lower probability of entering the CON membrane. In addition, mean square displacement (MSD) and diffusion coefficients analysis demonstrated substantially lower ionic mobility for Mg^2+^ relative to Li^+^ in both environments (Fig. 4, D to F). Free energy profiles for transmembrane migration were subsequently calculated, wherein the potential of mean force (PMF) quantitatively characterizes the energy barriers associated with ionic permeation (*9*, *56*). Analysis of PMF profiles demonstrated that Mg^2+^ (3.43 kcal mol^−1^) experiences a much higher energy barrier compared to Li^+^ (0.85 kcal mol^−1^), explaining its hindered transmembrane transport (Fig. 4G; see more details in movies S1 and S2). Collectively, the enhanced membrane partitioning ability and superior transport characteristics exhibited by Li^+^ relative to Mg^2+^ ultimately enable highly selective Li^+^/Mg^2+^ separation through the CON membrane.

In addition, simulations of Li^+^/Mg^2+^ separation were conducted at the chemical-microscopic molecular level using nonequilibrium MD (NEMD) simulations, which are recognized as an important means of studying the mechanisms of selective solute separation (*57*, *58*). Figure 4H illustrates the initial configuration and snapshots after 80 ns during the NEMD simulations. Throughout the process, the rate of water molecules through the CON membrane is relatively stable, while it is observed that Li^+^ successfully completes transmembrane transport, whereas Mg^2+^ is entirely rejected (Fig. 4I). This result is highly consistent with the experimentally observed trend and further confirms the efficient sieving ability of the CON membrane for Li^+^/Mg^2+^. By integrating the MD simulations, the ion-selective mechanism of the CON membrane was not only theoretically elucidated, but a robust microscopic explanation for the experiments was also provided.

### Process-scale analysis

Up to this point, most of the results above have been obtained and presented at the coupon scale, focusing on material-level performance, similar to many other studies reported in the literature (*59*, *60*). However, our recent findings reveal a clear discrepancy between coupon-scale performance and that observed at the module or process scale (the filtration setup at different scales can be found in fig. S11), which is more representative of real-world applications (*61*). Notably, high performance at the lab scale does not always translate into satisfactory outcomes at the process level. Therefore, this section investigates the critical process-scale performance of CON membranes through module-scale experiments and rigorous mathematical modeling.

**Fig. 5.**
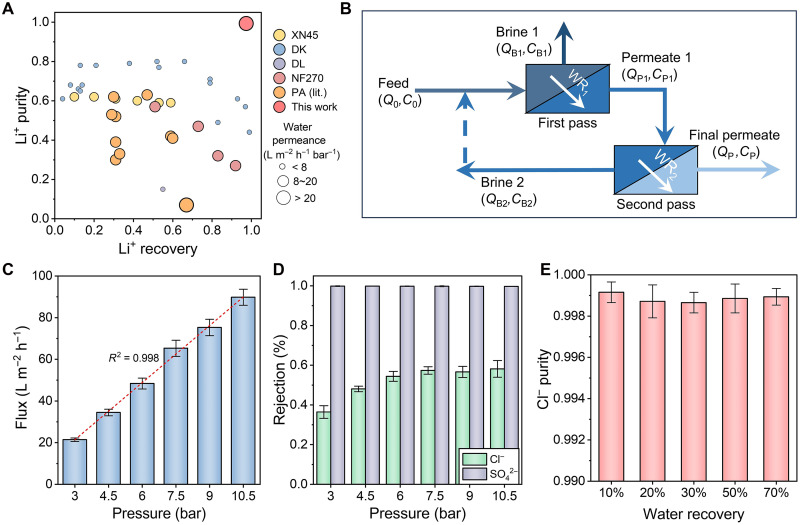
Process analysis of the ion sieving performance of CON membranes. (**A**) Performance comparison of various commercial and lab-fabricated nanofiltration membranes and the CON-T membrane in terms of Li^+^ purity and Li^+^ recovery. The symbol size reflects the water permeance (liter m^−2^ hour^−1^ bar^−1^) of the membranes, and different types of membranes are distinguished by color. It is worth noting that the data points presented in this work were obtained through module-scale experiments and rigorous mathematical modeling, while other data points were obtained from the literature. Detailed information can be found in table S7. (**B**) Schematic diagram of the two-pass process with brine recirculation for Li^+^/Mg^2+^ separation. The permeate of the first pass is the feed of the second pass, and the brine of the second pass recirculates back to the feed of the first pass (indicated by a dashed line). Similar to multipass seawater desalination processes (improve water purity), the multipass nanofiltration process can improve Li^+^ purity, while brine recirculation is used to reduce Li^+^ loss. Flux (**C**) and ion rejection (**D**) of the CON-I membrane under different pressures (3.0, 4.5, 6, 7.5, and 10.5 bar). The feed contained 500 ppm of Na_2_SO_4_ and 2500 ppm of NaCl, with a cross-flow velocity of 22.4 cm s^−1^, a test temperature of 25 ± 1°C, and a membrane area of 22.05 cm^2^. The permeate is recirculated back to the feed tank to maintain a stable feed concentration. (**E**) To better approximate real-world application scenarios, we adopted a module-scale performance testing approach to evaluate the purity of Cl^−^ under different water recovery levels (10, 20, 30, 50, and 70%). In (C) to (E), the error bars represent the standard deviation of data from at least three distinct samples.

We first examine the CON-T membrane (which achieved a balance between Li^+^ recovery and purity in pseudo–module-scale experiments; see fig. S12) for Li^+^/Mg^2+^ separation. Figure S13 confirms that our modeled results of CON-T membranes agree well with the experimental results in terms of Li^+^ recovery and Li^+^ purity for pseudo–module-scale experiments in batch mode, which further allows us to perform this two-pass system with recirculation design (Fig. 5B). The detailed methodology of this two-pass model can be found in text S3. Under optimal operational parameters, the CON-T membranes yielded record-high Li^+^ recovery (97.4%) and Li^+^ purity (99.3%) (Fig. 5A), notably surpassing literature benchmarks and commercial membrane performance. Despite these promising results, we acknowledge that the inherently high Mg^2+^/Li^+^ mass ratio in salt lakes makes it difficult to meet the stringent industrial Li^+^ purity requirement (98 to 99.9%) in a single-pass design. Crucially, our two-pass filtration system exceeds the Li^+^ purity achieved by commercial membranes after three to four passes (Fig. 5B) (*62*). In addition, DK membranes [widely recognized membranes for lithium extraction (*63*, *64*)] require three passes to match the performance of CON-T membranes on the basis of the same modeling approach (fig. S14 and table S8). This marked reduction in process complexity of the CON-T membrane could translate into substantial technical and economic advantages, including reduced land footprint, lower capital expenditure on equipment, and simplified production line management.

Strategically, the denser CON-I membrane was used for high-purity Cl^−^ extraction from simulated coal chemical wastewater. Rigorous pressure-change and concentration experiments were designed to validate its reliability under simulated production conditions (text S4). The CON-I membrane exhibited a robust, strongly linear correlation between flux and pressure (*R*^2^ > 0.998) (Fig. 5C) while simultaneously maintaining exceptional ion separation capability, evidenced by SO_4_^2−^ rejection exceeding 99.7% (Fig. 5D). Impressively, under module-scale testing, a Cl^−^ purity of 99.9% was achieved in single-pass filtration (Fig. 5E). In this regard, CON-I membranes demonstrate strong selectivity for SO_4_^2−^ ions. This capability is particularly valuable for mitigating scaling issues commonly encountered in high-salinity wastewater treatment (*27*, *65*). Furthermore, the selective removal of divalent ions enables salt fractionation, facilitating resource recovery and contributing to the overall sustainability and efficiency of the process.

**Fig. 6.**
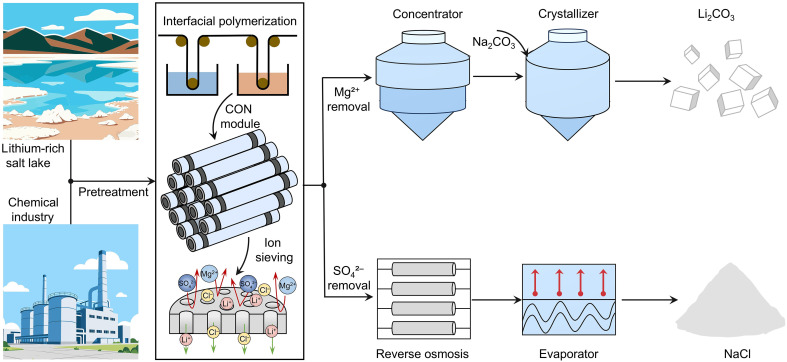
Potential applications of CON membranes. CON membranes are prepared via interfacial polymerization and can be scaled up to a membrane module format for seamless integration into existing roll-to-roll fabrication lines. The high permeability and ion selectivity of the membranes make them suitable for diverse applications, such as lithium extraction from salt-lake brines and industrial wastewater treatment. For lithium extraction, lithium-rich salt-lake brine undergoes pretreatment (e.g., particulate removal) before being processed through the CON module, where selective Mg^2+^ separation occurs, followed by concentration and crystallization to yield the Li_2_CO_3_ product. For NaCl recovery, following pretreatment, industrial wastewater is processed through the CON membrane module for selective SO_4_^2−^ removal, then concentrated via reverse osmosis, and crystallized through an evaporator to yield the NaCl product.

CON membranes, fabricated via the well-established IP technique, are compatible with existing manufacturing infrastructure. Exceptional water permeability coupled with high-precision ion separation capabilities enables CON membrane deployment in demanding separation processes. Key applications include selective Mg^2+^/Li^+^ separation for lithium extraction from salt-lake brines and targeted SO_4_^2−^ removal and recovery of Cl^−^ in industrial wastewater treatment (Fig. 6). CON membranes also show potential for heavy metal ion removal for groundwater treatment and seawater desalination pretreatment. These diverse applications highlight the versatility and scalability of CON membranes across multiple sectors. These findings underscore the potential of CON membranes to meet industrial demands for selective ion separation, resource recovery, and cost-effective process design.

## DISCUSSION

We have successfully prepared ultrathin and highly pore-connected CON membranes by the condensation reaction between cyclen and diacyl chloride. The fabrication process for CON membranes is highly compatible with that of commercial polymer membranes, without substantial alterations to existing production lines and infrastructure necessitated. The resulting CON membranes have high permeability and subångström ion resolution, capable of rapid and precise separation of both mono/divalent cations and anions. CON-T membranes enable efficient Li^+^ extraction from salt lakes (Li^+^ recovery: 97.4%; Li^+^ purity: 99.3%), and CON-I membranes enable high-purity Cl^−^ (purity ~99.9%) production from industrial wastewater. Our findings will advance the understanding of the construction of high-resolution ion separation membranes and offer technically practical solutions to address global challenges such as water scarcity and the energy transition.

## MATERIALS AND METHODS

### Materials and chemicals

PES (UE050), polyacrylonitrile (UN050), and polysulfone (US050) substrates were obtained from RisingSun Membrane Technology (Beijing) Co., Ltd.; detailed information on the PES substrate can be found in fig. S15 and table S9. Cyclen (98%) was purchased from Shanghai Tengqian Biological Technology Co., Ltd. TPC (99%), IPC (99%), trimesoyl chloride (99%), Na_2_SO_4_, MgSO_4_, MgCl_2_, BaCl_2_, CaCl_2_, NiCl_2_, CoCl_2_, KCl, LiCl, NaCl, *n*-hexane (analytical reagent), and *N*,*N*-dimethylformamide were obtained from Adamas-beta (China). SLS (99%), piperazine (99%), polyethylenimine [*M*_w_ (weight-average molecular weight) = 70,000 Da, 50% aqueous], polyethylene glycols (*M*_w_ = 200, 300, 400, and 600 Da), and diethylene glycol (106 Da) were purchased from Shanghai Macklin Biochemical Technology Co., Ltd.

### Fabrication of CON membranes

CON membranes were prepared by a well-established IP process (fig. S1, C and D). Typically, 0.5 g of cyclen and 0.027 g of SLS were dissolved in 100 ml of deionized (DI) water to prepare the aqueous phase, and 0.1 g of TPC or IPC was dissolved in 100 ml of *n*-hexane to prepare the organic phase. The PES substrate was first exposed to the aqueous phase and held for 3 min, followed by a rubber roller to remove the excess solution. Thereafter, an organic phase was applied to the amine monomer–saturated substrate to trigger IP reaction, and the reaction time was strictly controlled at 5 min. Once the organic phase was removed, the surface of the nascent membrane was thoroughly rinsed with fresh *n*-hexane to remove unreacted monomers. The fabricated CON membranes were lastly stored in DI water at 4°C.

### Characterization

Morphological characterization was performed using field emission SEM (ZEISS Sigma 300, Germany) to analyze both the surface topography and cross-sectional morphology of the membranes, with an accelerating voltage of 3 kV. Before morphological characterization, the samples were sputter-coated with a layer of gold using a Quorum SC7620 sputter coater. The coating was performed at a current of 10 mA for 60 s to achieve an approximate gold film thickness of 10 nm. Surface roughness and thickness measurements were conducted via AFM (Bruker Dimension Icon, Germany) under ambient conditions using tapping mode. For thickness analysis, the nonwoven support layer was excised and allowed complete dissolution of the PES matrix in *N*,*N*-dimethylformamide. Subsequently, the isolated CON selective layer was transferred to a silicon wafer. The STEM-EDX spectrum was obtained by STEM (JEM-F200, JEOL, Japan) equipped with a Super-X EDX detector. The surface chemical composition was analyzed through FT-IR (Thermo Fisher Scientific Nicolet iS20, US) and x-ray photoelectron spectroscopy (Thermo Fisher Scientific K-Alpha, US). Surface charge properties were assessed using a zeta potential analyzer (SurPASS3, Anton Paar, Austria) across pH 3 to 10, while wettability was quantified via water contact angle measurements (JC2000A, Shanghai Zhongchen Digital Technology Equipment Co., Ltd.) using 1 μl of ultrapure water droplets.

### Separation performance tests

Separation performance evaluation was conducted using a cross-flow filtration system featuring an effective membrane area of 22.05 cm^2^. Experiments were performed under controlled temperature (25 ± 1°C) with a cross-flow velocity of 22.4 cm s^−1^. All CON membranes were pre-pressurized using DI water at 6.0 bar for 2 hours. Subsequently, the feed solution was replaced with inorganic salts (1000 ppm) and neutral solutes (200 ppm) to assess the rejection of the CON membrane. The permeance (*P*, liter m^−2^ hour^−1^ bar^−1^) was obtained from Eq. 1

$$P=\frac{V}{A_{m}\times \text{Δt}\times \text{Δp}}$$ (1)

in which *V* represents the permeate volume, *A*_m_ denotes the area of filtration, ∆*t* indicates the filtration time specified, and ∆*p* is the pressure applied.

The rejection (*R*, %) was calculated by Eq. 2

$$R=\left(1-\frac{C_{p}}{C_{f}}\right)\times 100\%$$ (2)

where *C*_p_ and C_f_ represent the concentrations of the permeate and feed solution, respectively. A conductivity meter (DDS-11A, Leici, China) and total organic carbon analyzer (TOC-L CPH, Shimadzu, Japan) were used for determining the salt and neutral concentrations, respectively.

To evaluate the ion-sieving ability of the CON membranes, mixed salts were used as feed solutions. Specifically, for Li^+^/Mg^2+^, a binary mixture of MgCl_2_ and LiCl was used as the feed solution, and the total salt concentration and Mg^2+^/Li^+^ mass ratio were varied. For Cl^−^/SO_4_^2−^, a binary mixture of Na_2_SO_4_ and NaCl was used as a feed solution, controlling the Na_2_SO_4_/NaCl mass ratio of 1:1 and varying the total salt concentration. The selectivity [*S* (X/Y)] was evaluated by Eq. 3

$$S\;(X/Y)=\frac{1-R_{X}}{1-R_{Y}}$$ (3)

The concentration of cations was detected by inductively coupled plasma spectroscopy (Agilent 5110, US), and the concentration of anions was evaluated using ion chromatography (Thermo Fisher Scientific AQUION RFIC, US).

## Supplementary Materials

### The PDF file includes:

Supplementary Text Figs. S1 to S16 Tables S1 to S10 Legends for movies S1 and S2 References
