# Supplementary material for: Macrocycle-based covalent organic networks for ultrafast sub–1-Å precision ion sieving
Source: Sci Adv. 2026 Apr 17;12(16):eaed0804. doi: 10.1126/sciadv.aed0804 (PMC13089350; doi:10.1126/sciadv.aed0804)
Supplement: Supplementary file 1 — Supplementary Text Figs. S1 to S16 Tables S1 to S10 Legends for movies S1 and S2 References [file sciadv.aed0804_sm.pdf]

Science Advances
AAAS

Supplementary Materials for

Macrocycle-based covalent organic networks for ultrafast sub–1-Å precision ion sieving

Xiao-Gang Jin *et al.*

Corresponding author: Xiao-Hua Ma, xiaohuama@ecust.edu.cn; Zhe Yang, ceyangz@ust.hk

Sci. Adv. **12**, eaed0804 (2026)
DOI: 10.1126/sciadv.aed0804

The PDF file includes:

Supplementary Text
Figs. S1 to S16
Tables S1 to S10
Legends for movies S1 and S2
References

Other Supplementary Material for this manuscript includes the following:

Movies S1 and S2

Supplementary Text

Text S1 Determination of MWCO and pore size distribution of CON membranes

Consistent with our previous work(*41*, *66*), the pore size distribution and molecular weight cut off (MWCO) of the CON membranes were determined by the rejection rate of neutral organic molecules with different sizes. The neutral organic molecules used in this study include diethylene glycol (DEG, 106 Da), and different molecular weight polyethylene glycol (PEG, Mw = 200, 300, 400, and 600 Da). The feed concentration of each organic compound solution was 200 ppm. The test pressure was 6.0 bar, the temperature was 25 ± 1 °C, the cross-flow velocity was 22.4 cm s^{-1}, and the effective membrane area was 22.05 cm^2.

The MWCO is defined as the molecular weight of the neutral molecule at 90% retention. The pore size distribution curve is expressed as a probability density function, established under the assumption of no steric or hydrodynamic interaction between these neutral solutes and the membrane pores. The pore size distribution function is the following Eqs. (S1)~(S2):

$$ds = 33.46 \times 10^{-3} \times M_W^{0.557} \quad \text{(S1)}$$

$$\frac{dR\left(d_p\right)}{dd_p} = \frac{1}{d_p ln\sigma_p \sqrt{2\pi}} \exp\left[-\frac{(lnd_p - ln\mu_p)^2}{2(lnd_p)^2} \right] \quad \text{(S2)}$$

where M_W (Da) represents the molecular weight of PEG and DEG, σ_p represents the geometric standard deviation.

Text S2 Molecular dynamics simulations

CON Membrane modeling. The interfacial polymerization (IP) process for constructing covalent organic network (CON) membranes was modeled by initializing a system containing 100 1,4,7,10-tetraazacyclododecane (cyclen) molecules and 200 terephthaloyl chloride (TPC) or isophthaloyl chloride (IPC) molecules (**Fig. S16**). These monomers afford up to 400 potential reaction sites. Following geometric optimization of all monomers packed in a three-dimensional simulation cell, cross-linking was performed under the NVT ensemble. Amide bond formation with concomitant HCl generation occurred when the distance between the amino group nitrogen and acyl chloride carbon atoms fell below 3.5 Å. This reaction distance was incrementally increased to a 5.0 Å cutoff.

Upon cross-linking completion, residual HCl and unreacted monomers were removed. Subsequently, a 21-step equilibrium simulation was conducted to relax the CON membrane structure(**Table S10**)(*67*).

FFV and pore size distribution. Fractional free volume (*FFV*) of CON membrane was estimated by the following Eq. (S3):

$$FFV = \frac{V_f}{V_f + V_o} \times 100\% \tag{S3}$$

where V_f and V_o are the free volume and occupied volume. The values of V_f and V_o are explored using probe with radius of 1 Å, which can also give the morphology of voids in aggregate. The pore size distributions of CON-T and CON-I were calculated by Zeo++ software(*68*).

MSD and RDF analysis. The ion in bulk solution and CON membrane (CON-T) system were constructed respectively, with ion concentration of 0.5 M. After energy minimization, 5 ns NPT and 5 ns NVT ensemble simulations were performed for both systems. The temperature was controlled at 300 K using a Nose-Hoover thermostat, and the pressure was controlled at 1 bar using a Berendsen barostat. The last 1 ns of data from the NVT simulations were collected for analyses. The mean square displacement (*MSD*) and diffusion coefficient of ions (*D*) can be calculated using the Einstein relation, as Eqs. (S4)~(S5):

$$MSD = (r_j(t) - r_j(0))^2 = \frac{1}{N}\sum_{j=1}^{N}\left[(r_j(t) - r_j(0))^2\right] \tag{S4}$$

$$D = \frac{1}{6}\lim_{t\to\infty}\frac{dMSD(t)}{dt} = \frac{1}{6}\lim_{t\to\infty}\frac{d}{dt}\sum_{j=1}^{N}\left[(r_j(t) - r_j(0))^2\right] \tag{S5}$$

where *N* is the total number of the targeted ion, $r_j(0)$ and $r_j(t)$ are the position of molecule j at the time of the 0 and t, respectively.

The radial distribution functions (RDF, $g(r)$) was calculated to evaluate the hydration state of ions in bulk solution and in CON membrane. The number of water molecules around the ion (CN_r) was calculated using the following Eq. (S6):

$$CN_r = 4\pi\rho\int_0^r r^2 g(r)\,dr \tag{S6}$$

where ρ is the density of solution, r is the radial distance from the ion, and $g(r)$ is the radial distribution function at the distance r.

PMF calculation. The CON membrane system was extended to 10 nm along the Z-axis, and Li^+ /Mg^{2+} was placed in the system at coordinates (25 Å, 25 Å, 30 Å), filled with water molecules and balanced for charge with Cl^-. The system was further equilibrated for another 10 ns with semi-isotropic NPT simulations with the xy axis size fixed to equilibrate the water and ions. Finally, the potential of mean force (PMF) was calculated by umbrella sampling, pulling the ions along the z-axis through the CON membrane with a tensile constant of 1000 kJ mol^{-1} nm^{-2}. Each of the 27 replicas was sampled for 10 ns while taking the last 5 ns for analysis of the free energy using the g_wham program(*69*).

NEMD simulation. The system used for non-equilibrium molecular dynamics (NEMD) simulation contains two rigid graphene nanosheets to apply external pressure, a hydrated CON membrane, a feed chamber and a permeation chamber. The feed chamber contains 3000 water molecules, 25 Li^+, 25 Mg^{2+} and 75 Cl^- and the permeation chamber contains 1500 water molecules. The dimensions of the system were $3.93 \times 4.26 \times 25$ nm^3, with 5 nm for the CON membrane. After energy minimization of the system, a 1 ns equilibrium simulation was performed to compress the polymer membrane by applying a pressure of 1 bar on both sides of the piston to obtain a stable structure. Subsequently, an 80 ns NEMD was performed at an external pressure of 150 MPa.

PMF and NEMD simulations were performed using GROMACS 2020.6 software(*70*). The TIP3P model was used for water molecules, and OPLS-AA parameters were employed for other particles. The temperature was controlled using the Nosé-Hoover coupling method(*71*), and a 2.0 fs time step was utilized for integration. A cutoff length of 1.2 nm was applied to nonbonded interactions, and the particle mesh Ewald method with a Fourier spacing of 0.1 nm was employed for long-range electrostatic interactions(*72*). All covalent bonds to hydrogen atoms were constrained using the LINCS algorithm(*73*).

Text S3 Mathematical model for nanofiltration transport and process-scale modelling

DSPM-DE model for coupon-scale analysis. The simulation process in this work is based on our previous work using the well-established Donnan-steric pore model with dielectric exclusion

(DSPM-DE)(*61*, *74*). In brief, water and solute flux can be described by extended Nernst-Planck equation with the consideration of diffusion, convection, and electromigration effects (Eq. (S7))(*75*):

$$J_i = -K_{i,d} D_{i,\infty} \frac{dc_i}{dx} + K_{i,a} c_i J_v - \frac{z_i c_i K_{i,d} D_{i,\infty} F}{RT} \frac{d\psi(x)}{dx} \tag{S7}$$

where J_i is the solute flux, J_v is the water flux. c_i, and z_i are the concentration and valency of ion i, respectively. $K_{i,a}$ and $K_{i,d}$ are hindrance factors accounting for advection and diffusion, respectively. $D_{i,\infty}$ is the mass diffusivity in an infinitely diluted solution, and F and R are Faraday and ideal gas constants, respectively. T is the temperature, and $\psi(x)$ is the electrical potential at coordinate x inside the pore.

By assuming the continuity of Gibbs free energy at the solution-membrane interface, the ion concentration just inside the membrane ($c_{i,1}$) is derived from the feed-solution/membrane interface concentration ($c_{i,m}$), accounting for ion partitioning (Eq. (S8)):

$$\gamma_{i,1} c_{i,1} = \left(\Phi_S \Phi_D \Phi_{DE}\right) \times \gamma_{i,m} c_{i,m} \tag{S8}$$

where γ_i is the activity coefficient of ion i, and Φ_S, Φ_D, Φ_{DE} are the steric exclusion factor, Donnan exclusion factor, and dielectric exclusion factor, respectively.

Due to concentration polarization, the feed-solution/membrane interface concentration ($c_{i,m}$) is higher than the bulk solution concentration ($c_{i,f}$), given by Eq. (S9):

$$J_i = -k^{\bullet}_{c,i}\left(c_{i,m} - c_{i,f}\right) + J_v c_{i,m} - z_i c_{i,m} D_{i,\infty} \frac{F}{RT} \xi \tag{S9}$$

where $k^{\bullet}_{c,i}$ is the modified mass transfer coefficient incorporating the 'suction effect', and ξ is the electrical potential gradient at the feed-membrane interface.

Charge neutrality is applied to both the bulk solution and membrane pores to maintain chemical stability based on Eqs. (S10)~(S12):

$$\sum_i^n z_i c_{i,m} = 0 \tag{S10}$$

$$\sum_i^n z_i c_{i,p} = 0 \tag{S11}$$

$$\sum_{i}^{n} z_i c_i(x) + c_X = 0 \quad \text{(S12)}$$

where $c_{i,m}$ and $c_{i,p}$ are the concentrations of the feed-solution/membrane interface and the concentrations of permeate, $c_i(x)$ denotes the concentration of ion i at coordinate x inside the pore, and c_X denotes the average charge density of the membrane.

To apply DSPM-DE model, one requires two sets of inputs, i.e., membrane properties [membrane pore radius (r_p), effective membrane thickness (L_e), volumetric charge density (c_X), and relative permittivity inside membrane pores (ϵ_p)], and operation conditions [feed concentrations ($c_{i,f}$), water flux (J_v), and temperature (T)]. With these inputs, we can obtain permeate concentrations ($c_{i,p}$) and feed concentrations just outside the membrane ($c_{i,m}$) by numerically solving Eqs. (S7)~(S12)(*76*). More comprehensive description of the model, including its premises, governing equations, and solving methods, can be found in our previous work(*61*).

DSPM-DE model for module-scale analysis. To better reflect practical operation, the coupon-scale DSPM-DE model is further extended to module-scale via a one-dimensional finite element approach. The dimensions of the nanofiltration module in this study are adopted from our previous work(*61*). In brief, we adopted commercially available 8040-typed membrane module(*77*), where the effective leaf length (L_{module}) and width (W_{module}) are both 1.0 m, and the number of leaves (N_{leaf}) in the module is 20, where seven standard membrane modules are connected in series in a pressure vessel(*78*). To enable module-scale analysis, the full membrane sheet is discretized into K equal-area elements along the feed flow direction, each governed by the coupon-scale DSPM-DE model. Regarding the module-scale analysis, we focus on bulk feed concentrations, applied hydraulic pressure, and temperature, respectively. Consequently, local water flux (J_v) is obtained using the Hagen-Poiseuille equation as input for the DSPM-DE model (Eq. (S13))[9]:

$$J_v = \frac{r_p^2 \left(\Delta P - \Delta \pi_m\right)}{8 \mu L_e} \quad \text{(S13)}$$

where ΔP is the applied hydraulic pressure, $\Delta \pi_m$ is the osmotic pressure difference between feed and permeate, and μ is the dynamic viscosity of water, in which $\Delta \pi_m$ is estimated by the van't Hoff equation (Eq. (S14))[9]:

$$\Delta\pi_m = RT\sum_{i=1}^{n}\left(c_{i,m} - c_{i,p}\right) \tag{S14}$$

By coupling Eqs. (S13)~(S14) with the DSPM-DE model, water flux (J_v) can be iteratively solved from an initial guess, as it affects outputs of $c_{i,m}$ and $c_{i,p}$. Once J_v is determined, $c_{i,m}$ and $c_{i,p}$ are computed and the local feed concentration for the next differential element, $c_{i,f}^{loc}(k+1)$, is updated via mass conservation principles (Eqs. (S15) and (S16)).

$$Q_p\left(k\right) = Q_p\left(k-1\right) + J_v\left(k\right) \times W_{\text{module}} \times N_{\text{leaf}} \times dL \tag{S15}$$

$$c_{i,f}^{\text{loc}}\left(k+1\right) = \frac{\left(Q_0 - Q_p(k-1)\right) \times c_{i,f}^{\text{loc}}(k) - J_v(k) \times N_{\text{leaf}} \times W_{\text{module}} \times dL \times c_{i,p}^{\text{loc}}(k)}{Q_0 - Q_p(k)} \tag{S16}$$

Herein, Q_0, $Q_p(k)$, $J_v(k)$, $c_{i,f}^{loc}(k)$, and $c_{i,p}^{loc}(k)$ denote the volumetric feed flow rate of feed, the cumulative volumetric flow rate of permeate, local water flux, local feed concentration, and local permeate concentrate at k_{th} differential element, respectively. The membrane element is discretized into $K = L_{module}/dL$ elements of length dL. After completing the iteration, the cumulative permeate concentrations ($c_{i,p}^{cum}$) and water flow rate (Q_p^{cum}) can be obtained, from which the cumulative water recovery rate (WR) is calculated (Eq. (S17)):

$$WR = \frac{Q_p^{\text{cum}}}{Q_0} \tag{S17}$$

Since WR depends on ΔP, a MATLAB script was developed to compute the required ΔP for a target WR based on our previous work(*61*).

We simulate the module-scale Li^+/Mg^{2+} separation with the initial feed solution consists of a mixture of 4.61 mM LiCl and 26.74 mM $MgCl_2$, representing typical diluted brine with a magnesium-to-lithium mass ratio (MLR) of approximately 20. The feed flow rate (Q_0) is set to be 10 m^3 h^{-1} and the average water flux in the system is set to be approximately 25 L m^{-2} h^{-1}. Since we focus on module- scale performances, ion rejection varies throughout the membrane module due to changes in the local feed concentration. Species conservation is maintained in both the retentate and permeate by coupling adjacent differential elements, allowing the cumulative permeate concentration after k differential elements to be determined by Eq.(S18):

$$c_{i,p}^{\text{cum}}(k) = \frac{\int c_{i,p}^{\text{loc}}(k)\mathrm{d}k}{k} \tag{S18}$$

The cumulative rejection rate of ion i after k differential elements is subsequently calculated by Eq. (S19):

$$R_i^{\mathrm{cum}}(k) = 1 - \frac{c_{i,p}^{\mathrm{cum}}(k)}{c_{i,0}} \tag{S19}$$

where $c_{i,0}$ is the concentration of ion i in the initial feed solution. For example, when $k = 1$, $R_i^{\mathrm{cum}}(1)$ corresponds to the ion rejection rate of the first differential element, or WR ≈ 0. When $k = K$, $R_i^{\mathrm{cum}}(K)$ represents the cumulative ion rejection rate for the entire membrane module, where WR = 75% in this study.

With the ion rejection rates defined (Eq. (S19)), the widely-used membrane performance metric for Li^+/Mg^{2+} separation, separation factor $S_{\mathrm{Li/Mg}}$, is calculated via Eq. (S20):

$$S_{\mathrm{Li/Mg}}(k) = \frac{c_{\mathrm{Li,p}}^{\mathrm{cum}}(k) / c_{\mathrm{Mg,p}}^{\mathrm{cum}}(k)}{c_{\mathrm{Li,0}} / c_{\mathrm{Mg,0}}} = \frac{1 - R_{\mathrm{Li}}^{\mathrm{cum}}(k)}{1 - R_{\mathrm{Mg}}^{\mathrm{cum}}(k)} \tag{S20}$$

Similar to the case of cumulative rejection, when $k = 1$, $S_{\mathrm{Li/Mg}}(1)$ represents the separation factor of the first differential element, or WR ≈ 0. When $k = K$, $S_{\mathrm{Li/Mg}}(K)$ represents the total separation factor of the membrane module with a WR = 75% in this study.

In addition to the conventional metric $S_{\mathrm{Li/Mg}}$, this study also evaluates nanofiltration membrane performance using another two robust criteria: Li^+ purity (η_{Li}) and Li^+ recovery (LiR)(*45*). Li^+ purity is the mass fraction of Li^+ among cations in the permeate, and is directly related to $S_{\mathrm{Li/Mg}}$ (Eq. (S21)):

$$\eta_{\mathrm{Li}} = \frac{c_{\mathrm{Li,p}}^{\mathrm{cum}}}{c_{\mathrm{Li,p}}^{\mathrm{cum}} + c_{\mathrm{Mg,p}}^{\mathrm{cum}}} = \frac{1}{1 + \frac{\mathrm{MLR}}{S_{\mathrm{Li/Mg}}}} \tag{S21}$$

On the other hand, Li^+ recovery is used to evaluate the treatment efficiency, which is defined as the ratio of Li^+ in the final permeate to that in the feed solution (Eq. (S22)):

$$\mathrm{LiR} = \frac{Q_{\mathrm{p}}^{\mathrm{cum}} c_{\mathrm{Li,p}}^{\mathrm{cum}}(K)}{Q_0 c_{\mathrm{Li,0}}} = \mathrm{WR}\left(1\text{-}R_{\mathrm{Li}}^{\mathrm{cum}}(K)\right) \tag{S22}$$

where $c_{\mathrm{Li,0}}$ is the concentration of Li^+ in the feed solution.

To assess the DSPM-DE model's accuracy at the module-scale, the predictions were compared with experimental results obtained using CON-T membranes. Filtration experiments were conducted in a lab-scale cross-flow setup, with an effective membrane area of 22.05 cm^2 and cross-flow velocity of 22.4 cm s^{-1}. Initially, coupon-scale experiments with negligible water recovery were performed to determine membrane parameters. The feed solution was a binary salt mixture containing 4.61 mM LiCl and 26.74 mM $MgCl_2$, which was identical to the inputs for our module-scale simulation. The concentrations of Li^+ and Mg^{2+} in the feed and permeate solutions were quantified using an inductively coupled plasma optical emission spectrometry (ICP-OES) at varying water flux at different hydraulic pressures (i.e., 2, 3, 4.5, 5, 6, 7.5, and 9.0 bar), and Cl^- concentrations were determined based on the electroneutrality condition, as shown in **Fig. S13** Through these experiments with charged species, four membrane parameters (r_p, L_e, c_X and ϵ_p) were simultaneously determined using a global optimization method(*79*), resulting in regressed values of 0.49 nm, 2.03 μm, -0.24 mol m^{-3} and 31.64, respectively, for the CON-T membrane. Pseudo-module-scale experiments in batch mod were then performed to evaluate Li^+ purity and Li^+ recovery under different water recovery (10%, 20%, 30%, 50%, and 70%) at a fixed hydraulic pressure of 4.5 bar (see **Fig. S13**). Specifically, the brine stream was circulated back to the feed tank, and the permeate stream was collected in a separate tank until the target water recovery was achieved. During our pseudo-module-scale experiment, a feed solution of ~ 5 mL was sampled at the beginning of each filtration and permeate solutions were sampled at different water recovery with a sampling volume of ~ 5 mL per sample. **Fig. S13** presents a good agreement between experimental data and model predictions. These results allow us to further perform the process-scale analysis of CON membranes for Li^+/Mg^{2+} separation.

Modeling multi-pass nanofiltration with Brine Recirculation for CON-T membrane. We simulate the process scale Li^+/Mg^{2+} separation using a multi-pass nanofiltration system with brine recirculation for CON-T membrane based on the literature(*62*). The only difference is that we adopted the DSPM-DE model, which is physically grounded and well-established, instead of the solution-diffusion–electromigration (SDEM) model used in Wang's work.

Similarly, the initial feed solution consists of a mixture of 4.61 mM LiCl and 26.74 mM $MgCl_2$, which was identical to the inputs for our module-scale simulation. The feed flow rate (Q_0) is set to be 10 m^3 h^{-1} and the average water flux in the system is set to be approximately 25 L m^{-2}

h^{-1}. The performance of multi-pass nanofiltration with brine recirculation was simulated using a modeling approach.

For multi-pass filtration without brine recirculation, each pass can be solved sequentially with the permeate composition of one pass used as the feed to the next pass (**Fig. 5B**). For multi-pass filtration with brine recirculation, the feed of each pass between the first and last pass (i.e., second to ($N-1$)th pass) is a mixture of the permeate from the previous pass and the brine from the next pass. When steady-state operation is achieved, the mass balance of water can be described as Eq. (S23):

$$Q_n = \begin{cases} Q_0 + Q_2\left(1 - WR_2\right), for n = 1 \\ Q_{n-1}WR_{n-1} + Q_{n+1}\left(1 - WR_{n+1}\right), for n = 2 \sim N-1 \\ Q_{n-1}WR_{n-1}, for n = N \end{cases} \tag{S23}$$

Where N is the number of passes, Q_n and WR_n are the feed flow rate and water recovery of pass n, respectively, and Q_0 is the initial feed flow rate. Q_n is a function of Q_0 and the water recovery of each pass and can be solved analytically. The mass balance of ions can be described as Eq. (S24):

$$c_{f,n} = \begin{cases} Q_0c_0 + Q_2\left(1 - WR_2\right)c_{b,2}, for n = 1 \\ Q_{n-1}WR_{n-1}c_{p,n-1} + Q_{n+1}\left(1 - WR_{n+1}\right)c_{b,n+1}, for n = 2 \sim N-1 \\ c_{p,n-1}, for n = N \end{cases} \tag{S24}$$

where $c_{f,n}$, $c_{p,n}$, and $c_{b,n}$ are feed, permeate, and brine concentrations of pass n. $c_{f,n}$ depends upon both $c_{p,n-1}$ and $c_{b,n+1}$ and is thus solved iteratively until a steady state is found. For the CON-T membrane, a two-pass with recirculation configuration was adopted, as it already achieves excellent Li^+ purity and recovery. Detailed information on water recovery and applied pressure for each pass is provided in **Table S9**.

To enable a fair comparison, we also evaluated the performance of the commercial DK membrane (GE Osmonics, Minnetonka, MN), which is considered one of the most effective commercial membranes for Li^+/Mg^{2+} separation(*46*, *63*, *64*, *80*). For the DK membrane, both two-pass and three-pass configurations with brine recirculation were examine. The two-pass setup showed unsatisfactory performance, while the three-pass configuration improved separation but introduced additional process complexity and increased capital and membrane costs, making it

less desirable (**Fig. S14**, and **Table S9**). These results further substantiate the superiority of the CON-T membrane in achieving highly efficient Li^+/Mg^{2+} separation.

Text S4 Coupon-scale and pseudo-module-scale Cl^-/SO_4^{2-} separation experiments

For Cl^-/SO_4^{2-} system, the feed solution comprised a binary salt mixture of 500 ppm Na_2SO_4 and 2500 ppm NaCl, simulating typical coal chemical wastewater. Pseudo-module-scale experiments were performed for CON-I membrane at a fixed hydraulic pressure of 6.0 bar. The teste temperature was 25 ± 1 °C, the cross-flow velocity was 22.4 cm s^{-1}, and the effective membrane area was 22.05 cm^2.

Supplementary Figures

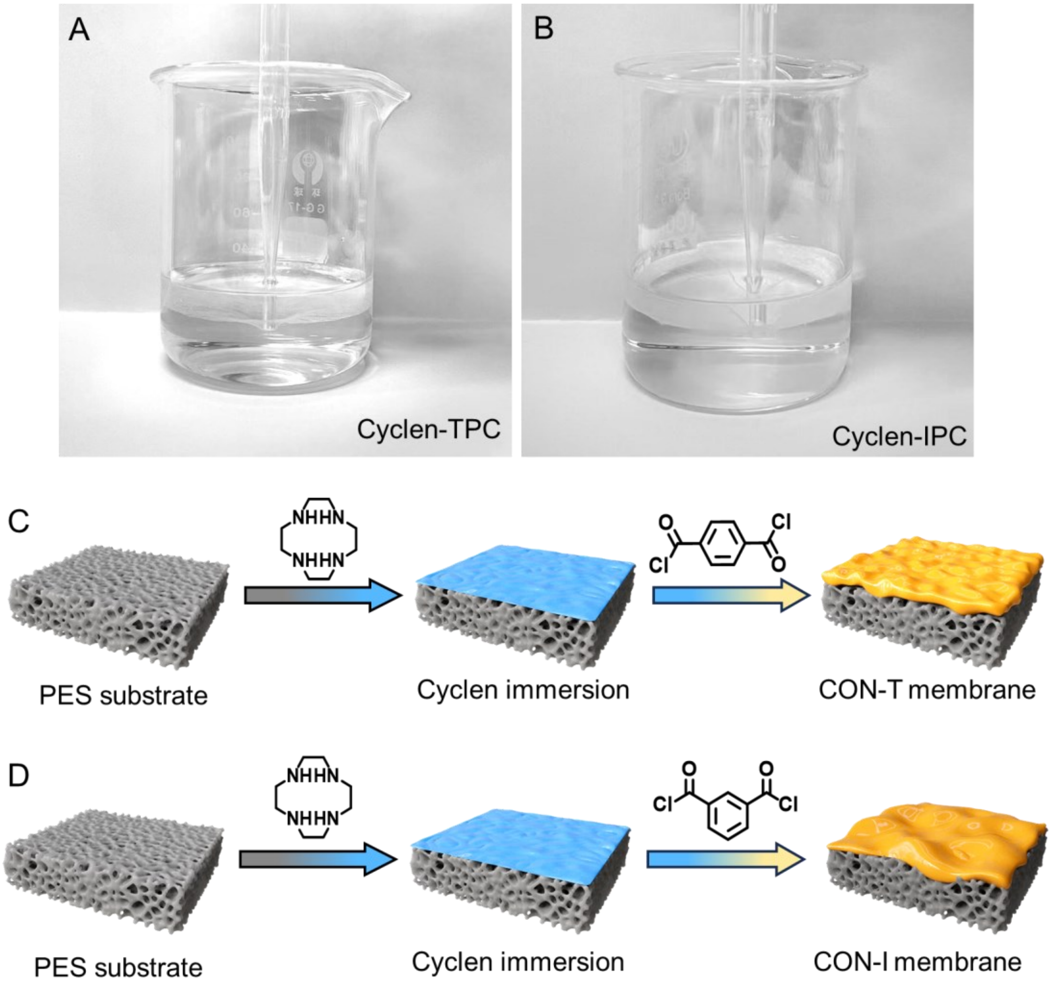

Fig. S1. Synthesis of CON membranes. (A, B) Optical images of free-standing films formed by cyclen and TPC (A) or IPC (B). Aqueous phase (cyclen concentration, 0.5 w/v%, SLS concentration, 1 mM), organic phase (TPC/IPC concentration, 0.1w/v%), reaction time, 5min. (C, D) Schematic of CON-T (C) and CON-I (D) membranes preparation using in situ interfacial polymerization.

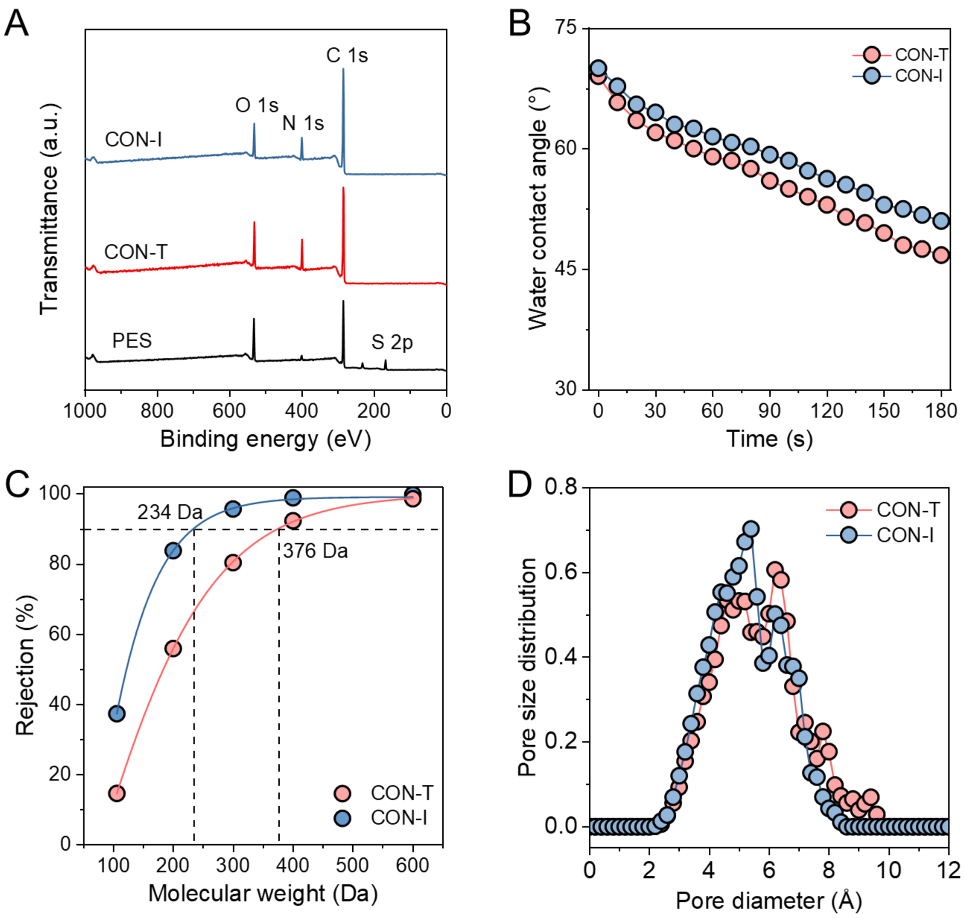

Fig. S2. Physicochemical properties of CON membranes. (A) XPS spectra, the N 1s peaks of the CON-T and CON-I membranes confirmed the formation of selective layer on PES substrate. (B) Water contact angle of CON-T and CON-I membranes. (C) MWCO of CON-T and CON-I membranes. The feed concentration of each organic compound solution was 200 ppm. The test pressure was 6.0 bar, the temperature was 25 ± 1 °C, the cross-flow velocity was 22.4 cm s^{-1}, and the effective membrane area was 22.05 cm^2. (D) Pore size distributions of CON-T and CON-I membranes obtained by MD simulations.

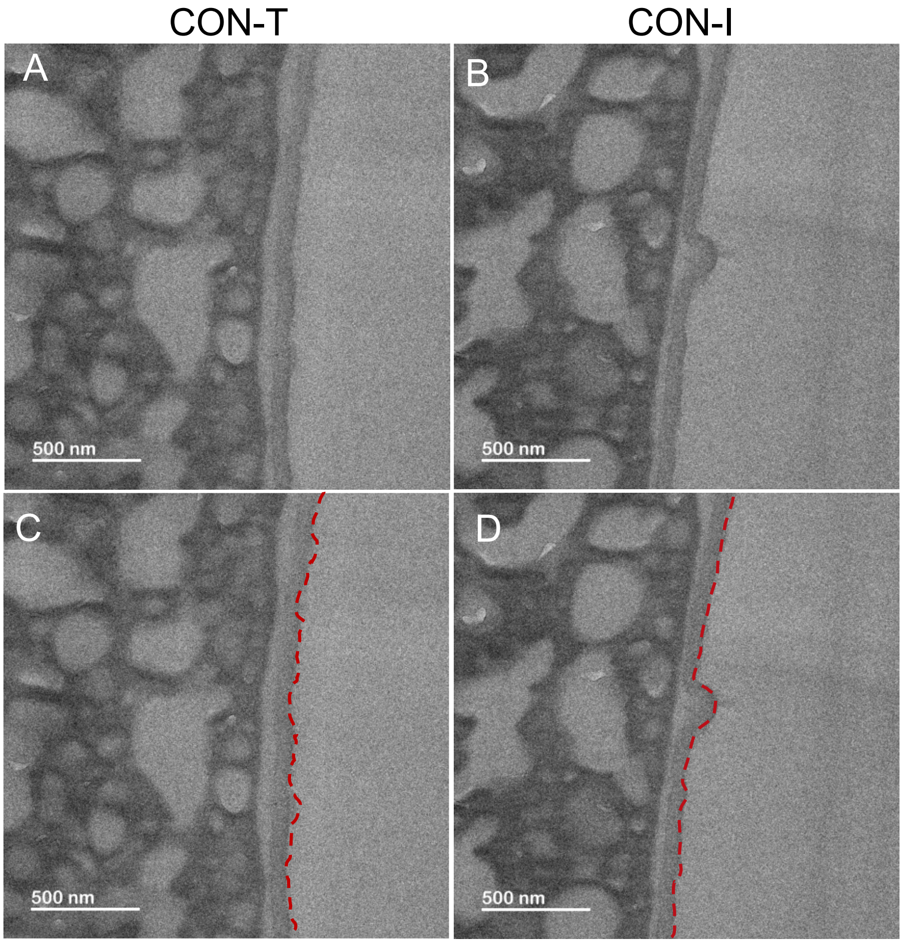

Fig. S3. Low-magnification TEM images of CON-T (A & C) and CON-I (B & D) membranes cross-sections. (A, B) Original TEM image, (C, D) adds red dashed lines to indicate the undulations on the membrane surface.

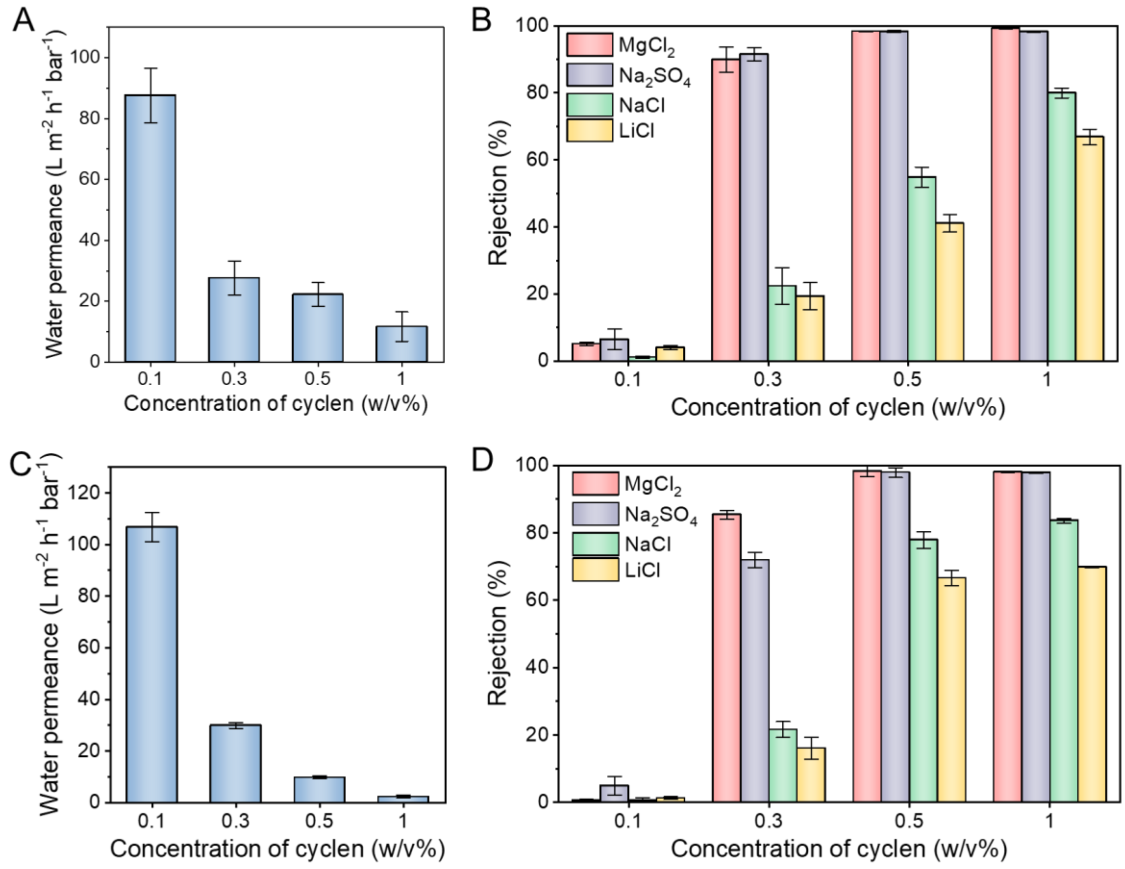

Fig. S4. Effect of cyclen concentration on the permeation selectivity of CON membranes. (A, B) Water permeance and salts rejection of CON-T membranes. (C, D) Water permeance and salts rejection of CON-I membranes. Reaction time, 5 min, SLS concentration, 1 mM. The feed concentration of each salt solution was 1000 ppm. The test pressure was 6.0 bar, the temperature was 25 ± 1 °C, the cross-flow velocity was 22.4 cm s^{-1}, and the effective membrane area was 22.05 cm^2. The data are presented as the mean ± standard deviation (SD) of membrane performance parameters from at least three independent experiments.

As the cyclen concentration increases, the water permeance gradually decreases and the salt rejection gradually increases. A preferred cyclen concentration is 0.5 w/v%.

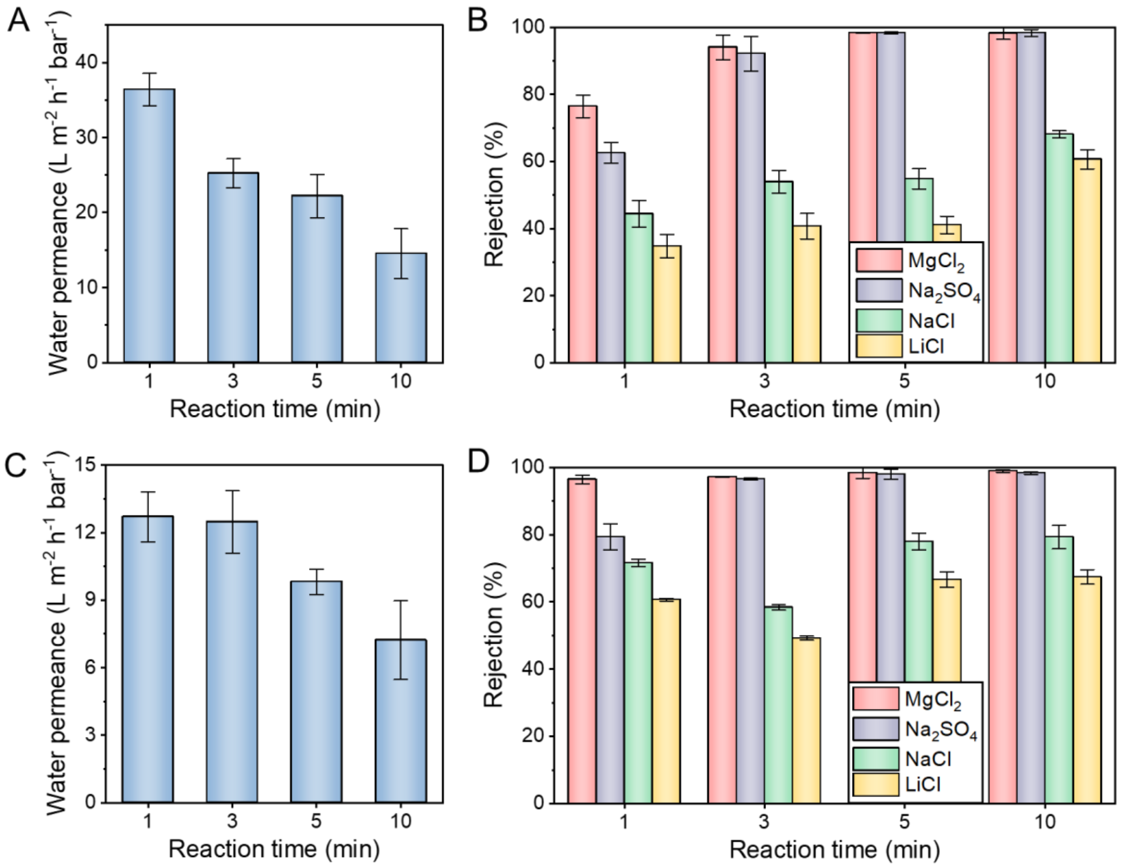

Fig. S5. Effect of reaction time on the permeation selectivity of CON membranes. (A, B) Water permeance and salts rejection of CON-T membranes. (C, D) Water permeance and salts rejection of CON-I membranes. cyclen concentration, 0.5 w/v%, SLS concentration, 1 mM. The test pressure was 6.0 bar, the temperature was 25 ± 1 °C, the cross-flow velocity was 22.4 cm s^{-1}, and the effective membrane area was 22.05 cm^2. The data are presented as the mean ± SD of membrane performance parameters from at least three independent experiments.

As the reaction time increases, the water permeance gradually decreases and the salt rejection gradually increases. A preferred reaction time is 5 min.

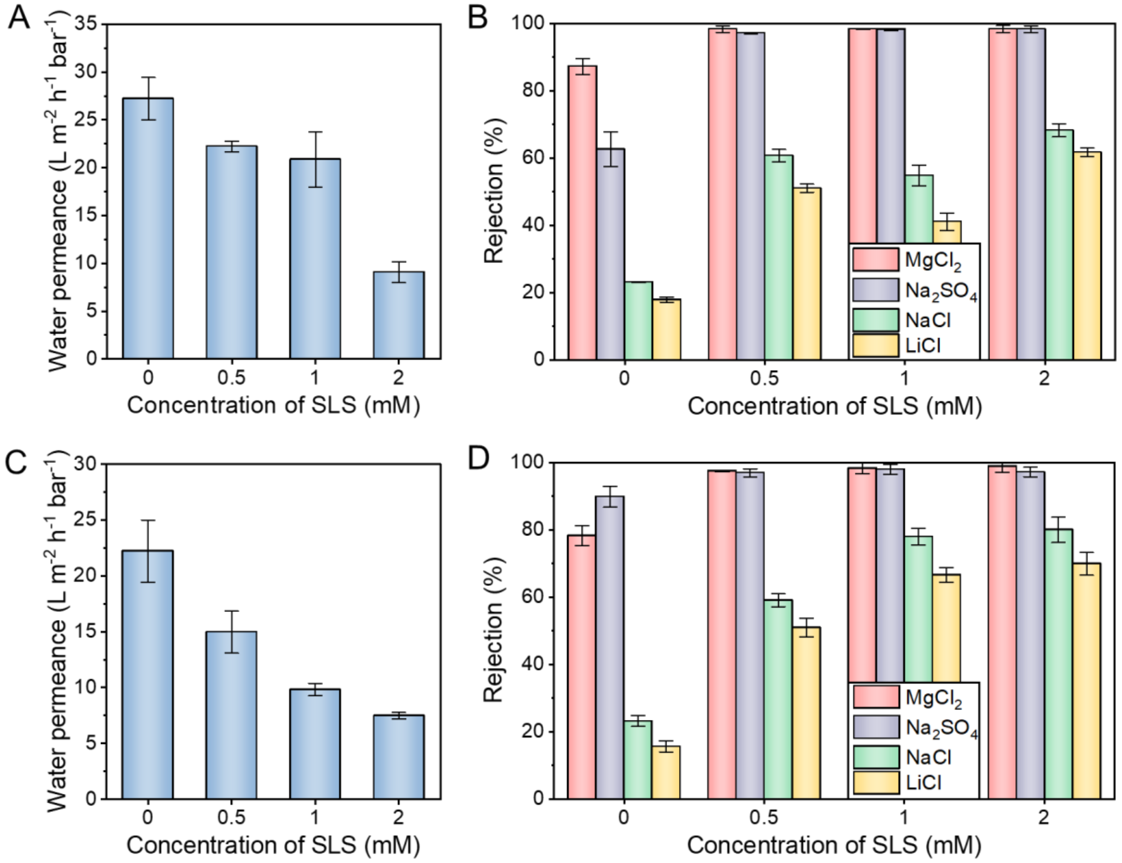

Fig. S6. Effect of SLS concentration on the permeation selectivity of CON membranes. (A, B) Water permeance and salts rejection of CON-T membranes. (C, D) Water permeance and salts rejection of CON-I membranes. Reaction time, 5 min, cyclen concentration, 0.5 w/v%. The test pressure was 6.0 bar, the temperature was 25 ± 1 °C, the cross-flow velocity was 22.4 cm s^{-1}, and the effective membrane area was 22.05 cm^2. The data are presented as the mean ± SD of membrane performance parameters from at least three independent experiments.

As the SLS concentration increases, the water permeance gradually decreases and the salt rejection gradually increases. A preferred SLS concentration is 1 mM.

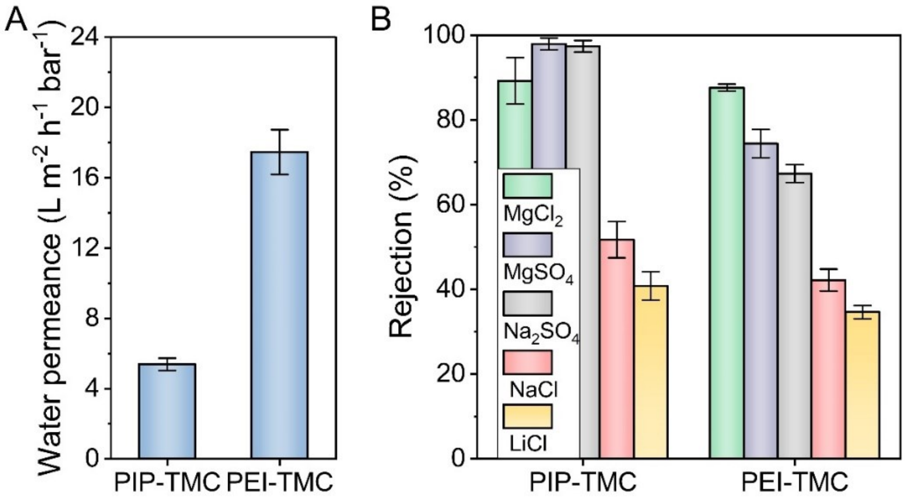

Fig. S7. Water permeance and inorganic salt retention of CON membranes prepared with TMC as the organic phase monomer. The reaction conditions were fixed as follows: a reaction time of 5 min, a PIP or PEI concentration of 0.5 w/v%, an SLS concentration of 1 mM, and a TMC concentration of 0.1 w/v%. The filtration performance was evaluated under the following conditions: a pressure of 6.0 bar, a temperature of 25 ± 1 °C, a cross-flow velocity of 22.4 cm s^{-1}, and an effective membrane area of 22.05 cm^2. All data for membrane performance parameters are presented as the mean ± SD from a minimum of three independent experiments.

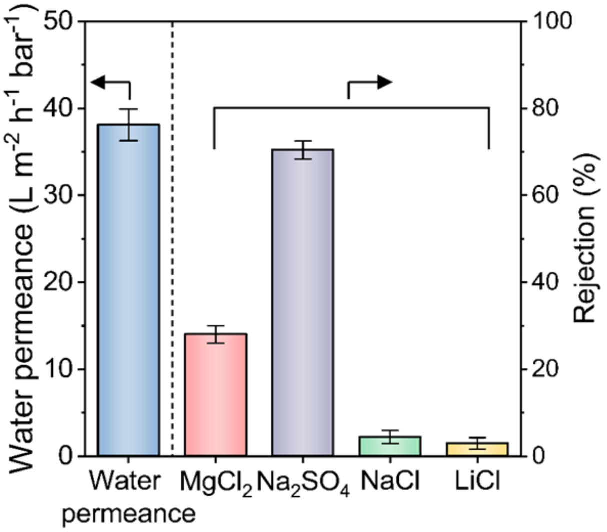

Fig. S8. Water permeance and inorganic salt rejection of CON membranes prepared with TMC as the organic phase monomer. The reaction conditions were fixed as follows: a reaction time of 5 min, a cyclen concentration of 0.5 w/v%, an SLS concentration of 1 mM, and a TMC concentration of 0.1 w/v%. The filtration performance was evaluated under the following conditions: a pressure of 6.0 bar, a temperature of 25 ± 1 °C, a cross-flow velocity of 22.4 cm s^{-1}, and an effective membrane area of 22.05 cm^2. All data for membrane performance parameters are presented as the mean ± SD from a minimum of three independent experiments.

Results indicate that CON membranes prepared with TMC as the organic phase monomer exhibit higher permeability but limited rejection of inorganic salts (**Fig. S8**). This performance difference primarily stems from the functionality and molecular structure of the monomers. TMC is a trifunctional monomer with three symmetrically arranged acyl chloride groups. Its high reactivity facilitates the formation of a cross-linked network during rapid interfacial polymerization, but may be less conducive to forming a selective layer with uniform and sufficiently dense pore sizes. In contrast, as difunctional monomers, TPC and IPC, when precisely controlled within the CON polymerization platform in this study, can form a polyamide layer with higher cross-linking density and a more homogeneous and dense network structure. This enables precise sieving of ions based on size while maintaining reasonable water permeability, constituting their core advantage over the classic TMC for this specific application.

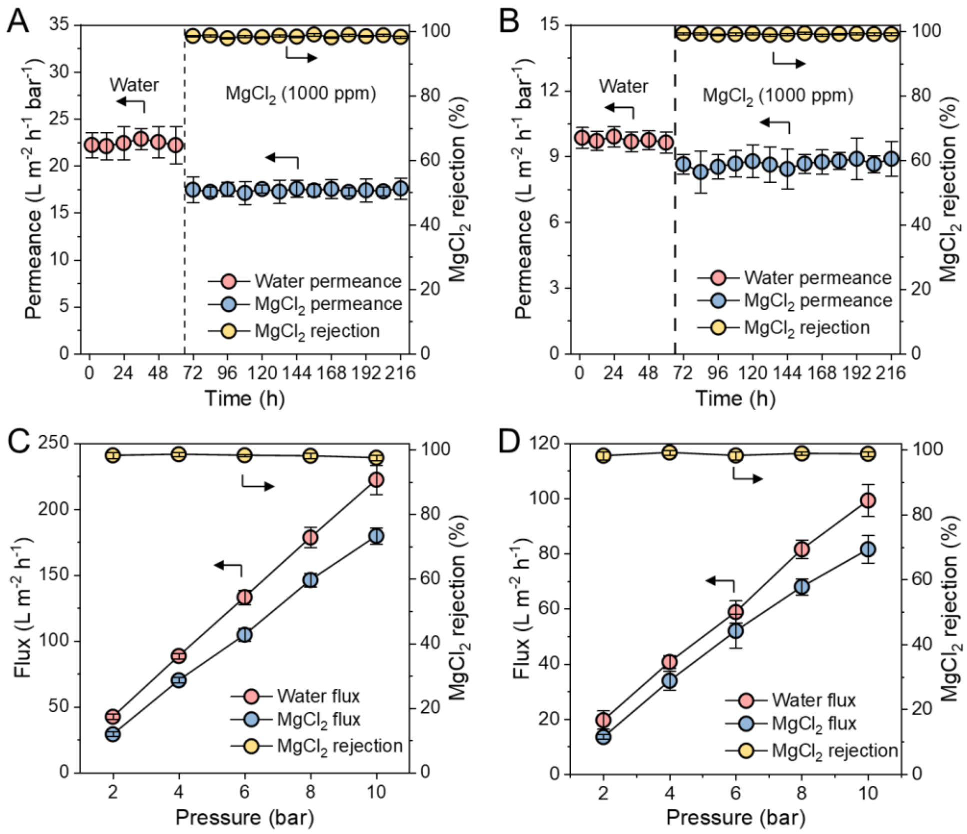

Fig. S9. Long-term stability and operational flexibility of CON membranes. (A, B) Permeability and $MgCl_2$ retention capacity of CON-T (A) and CON-I (B) membranes in long-term continuous operation (6.0 bar). (C, D) Flux and $MgCl_2$ rejection of CON-T (C) and CON-I (D) membranes at different operating pressures (2.0, 4.0, 6.0, 8.0, and 10.0 bar). The temperature was 25 ± 1 °C, the cross-flow velocity was 22.4 cm s^{-1}, and the effective membrane area was 22.05 cm^2. The data are presented as the mean ± SD of membrane performance parameters from at least three independent experiments.

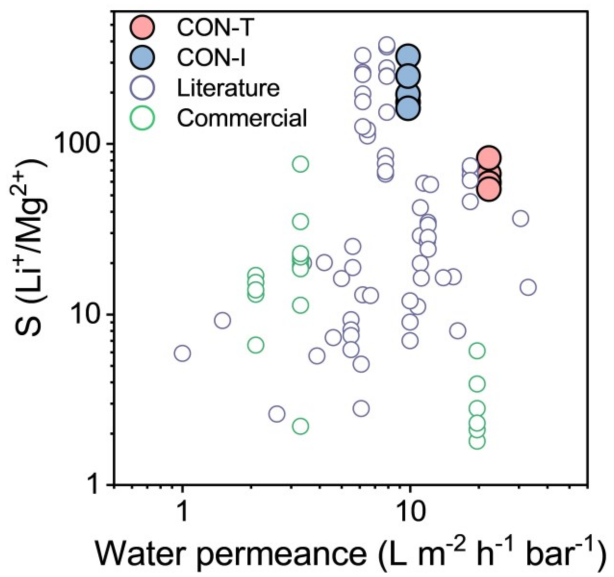

Fig. S10. Comparison of Li^+/Mg^{2+} separation performance based on water permeance and Li^+/Mg^{2+} selectivity.

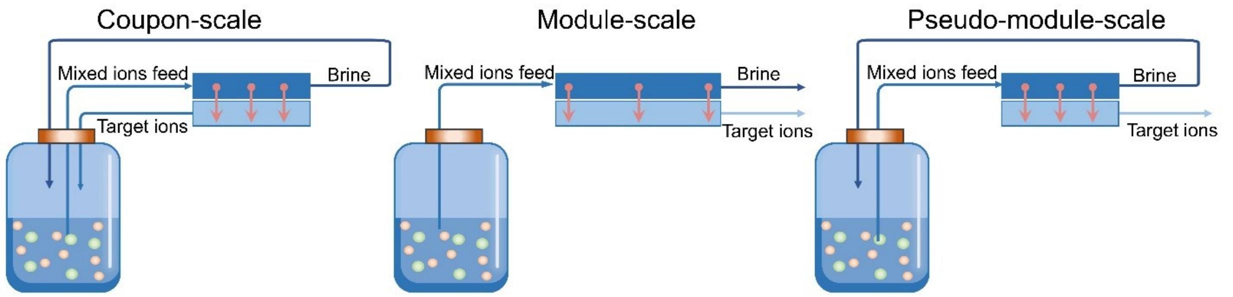

Feature	Coupon-scale	Module-scale	Pseudo-module-scale
Membrane area	Small	Large	small
Permeate back to feed tank	Yes	No	Yes
Spatial distribution	Negligible	Yes	Negligible
Temporal change	No	No	Yes
Feed tank concentration	Constant	Constant	Time dependent
Feed channel concentration	Constant	Position dependent	constant
Water flux	Constant	Position dependent	Time dependent

Fig. S11. Coupon-scale, module-scale, and pseudo-module-scale filtration setup. These schematics adopted an evaluation framework similar to the one published by Lin(*81*).

Coupon-scale experiments are performed using a small membrane area with a relatively large feed flow rate. The permeate is sent back to the feed tank, except for a small volume that is sampled for analysis, to maintain a constant feed composition. With coupon experiments, it can be assumed that the feed channel composition and water flux in the test cell are spatiotemporally constant, assuming no fouling occurs. Module-scale experiments are performed with a large membrane area, as in practical desalination plants. Using a module with a large membrane area induces spatial distribution of composition in the feed channel and water flux. Given the unavailability of pilot-scale filtration equipment, operating a coupon-scale system without permeate recycle (pseudo-module-scale experiments) enables the extraction of module-scale information. Coupon-scale experiments are used to understand the performance of membrane materials under specific feed compositions and applied pressures. Module-scale or pseudo-module-scale experiments are more suitable for studying the system-level behavior of membrane modules in actual resource recovery processes.

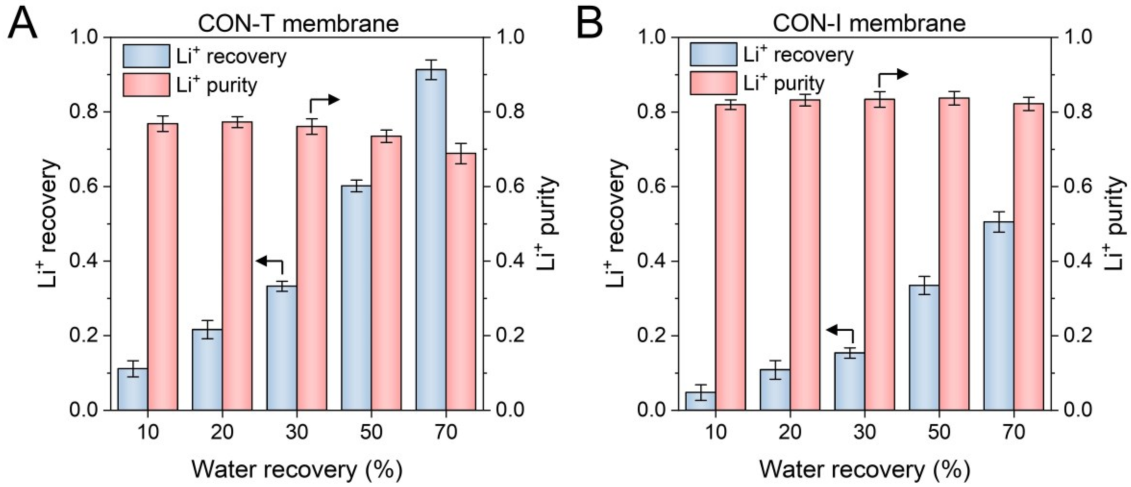

Fig. S12. Comparison of Li^+ purity and recovery for the CON-T (A) and CON-I (B) membranes in pseudo-module-scale experiments. The temperature was maintained at 25 ± 1 °C, and the cross-flow velocity was set to 22.4 cm s^{-1}. The initial water flux was fixed at 40 L m^{-2} h^{-1} under feed conditions of 4.61 mM Li^+ and 26.74 mM Mg^{2+}. The effective membrane area was 22.05 cm^2. The experiment data are presented as the mean ± SD of membrane performance parameters from at least three independent experiments.

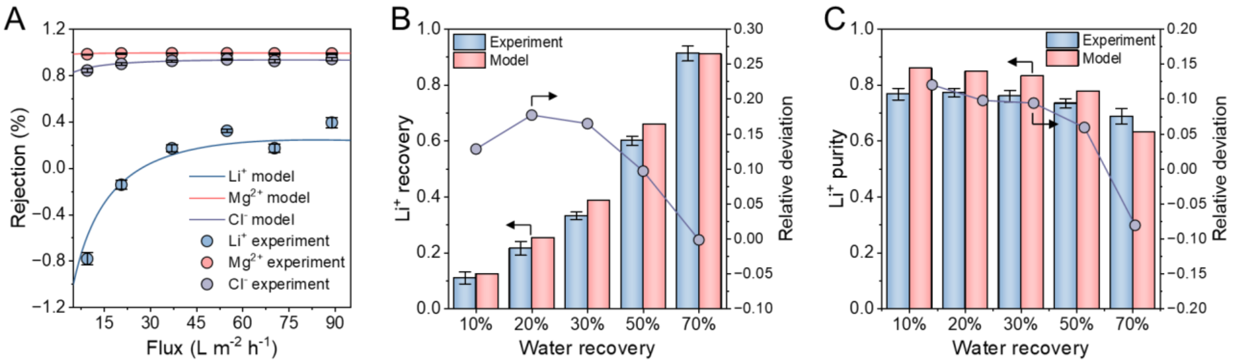

Fig. S13. Consistency analysis between experiments and simulations. (A) Coupon-scale experimental results and modelled predictions of ion rejections under different water flux at Li^+ concentration of 4.61 mM and Mg^{2+}concentration of 26.74 mM. Comparison of Li^+ purity (B) and recovery (C) data obtained in pseudo-module-scale experiments and predicted by the model under a hydraulic pressure of 4.5 bar. The test temperature was 25 ± 1 °C, the cross-flow velocity was 22.4 cm s^{-1}, and the effective membrane area was 22.05 cm^2. The experiment data are presented as the mean ± SD of membrane performance parameters from at least three independent experiments.

As shown in **Fig. S13**, the experimental values fit the model well, with small relative deviations.

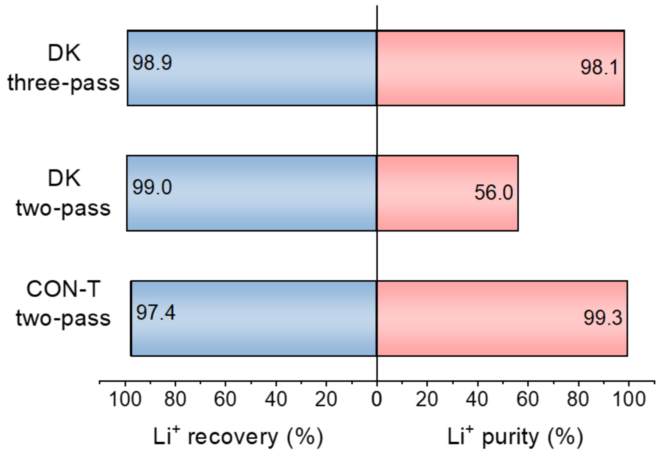

Fig. S14. Comparison of Li^+ recovery and purity obtained by multi-pass nanofiltration using CON-T and DK membranes. The water recovery for one-pass is set to 95%, the water recovery rate for two-pass is set to 85%, and the water recovery rate for three-pass is set to 85% (only for DK membrane).

As shown in **Fig. S14**, CON-T membrane outperforms DK membrane in two-pass with recirculation and has similar performance compared to DK membrane using three-pass with recirculation. Therefore, CON-T membrane requires less passes with lower capital cost and less process complexity.

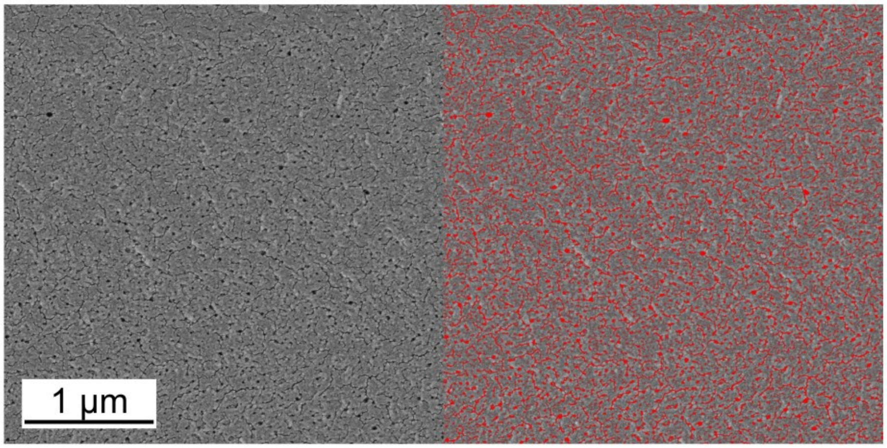

Fig. S15. SEM image of the PES substrate. The left side shows the original image, while the right side highlights the pore regions of the PES substrate in red.

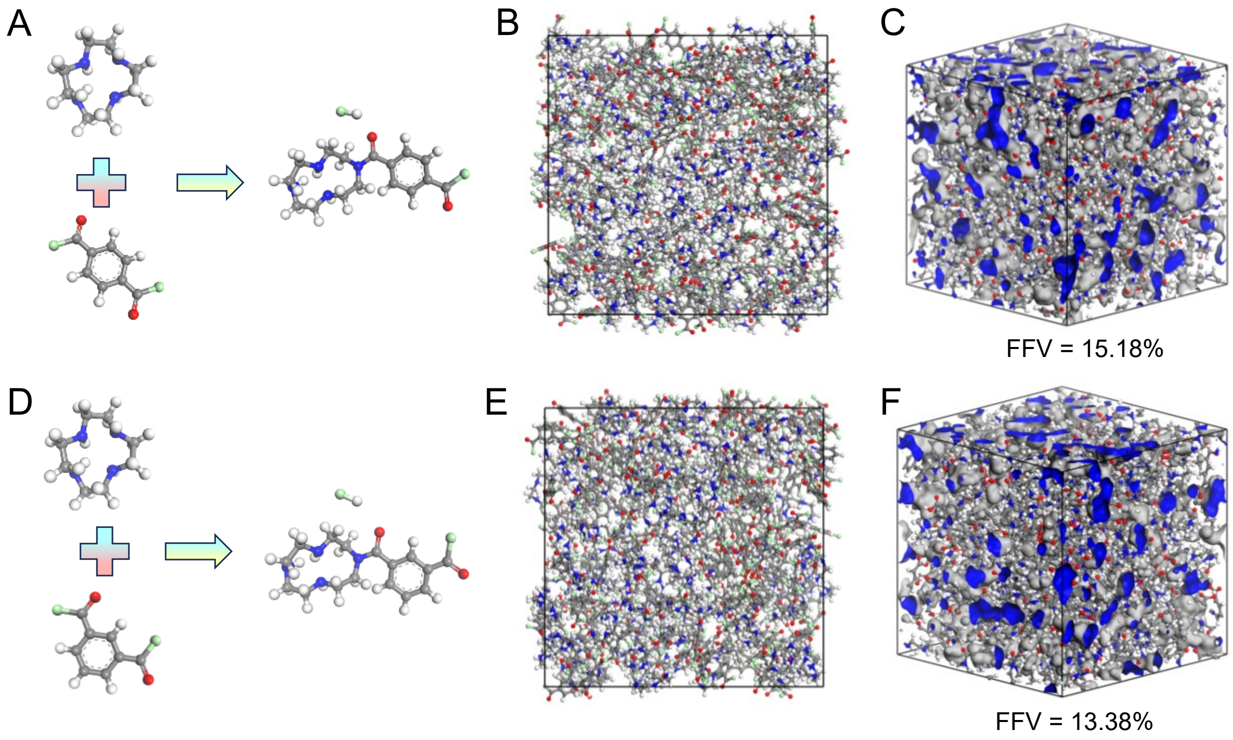

Fig. S16. Modeling of CON membranes. (A, D) Chemical structures for two reacted monomers, and the cross-linking reaction forming the CON-T (A) and CON-I (D) membranes, respectively. (B, E) Snapshot of systems at the beginning of the cross-linking process of CON-T (B) and CON-I (E) systems, respectively. (C, F) Snapshot of the final CON-T (C) and CON-I (F) membranes model, respectively. The blue region represents the free volume of the membrane, and the gray region indicates the Connolly surface of the membranes.

Supplementary Tables

Table S1. Elemental compositions measured by XPS.

Membranes	Atom percent (%)			
	O 1s	N 1s	C 1s	S 2p
PES substrate	22.26	\	71.68	6.06
CON-T	14.34	12.6	73.06	\
CON-I	11.01	10.47	78.53	\

Table S2. Water permeance and salts rejection of CON membranes prepared on different substrates.

Substrate	Membrane	Water permeance (L m^{-2} h^{-1} bar^{-1})	Rejection (%)			
			$MgCl_2$	Na_2SO_4	LiCl	NaCl
PES	CON-T@PES*	22.2±2.2	98.3±0.3	98.2±0.3	41.1±2.6	54.8±3.0
PES	CON-I@PES*	9.8±0.5	99.2±0.3	98.9±1.2	66.61.8	77.9±2.0
PAN	CON-T@PAN	13.8±0.9	91.8± 0.4	92.8±0.1	42.9±1.3	52.9±1.2
PAN	CON-I@PAN	8.5±1.2	96.8±0.3	97.0±0.1	60.6±2.0	72.1±3.3
PSF	CON-T@PSF	7.9±0.3	97.9±0.2	97.7±0.3	68.2±1.0	77.0±0.9
PSF	CON-I@PSF	4.2±0.4	96.5±0.4	96.6±0.2	73.8±2.1	80.3±0.6

：CON-T@PES and CON-T@PES* correspond to CON-T and CON-I membranes in the manuscript, respectively.

The reaction conditions were fixed as follows: a reaction time of 5 min, a cyclen concentration of 0.5 w/v%, an SLS concentration of 1 mM, and a TPC or IPC concentration of 0.1 w/v%. The filtration performance was evaluated under the following conditions: a pressure of 6.0 bar, a temperature of 25 ± 1 °C, a cross-flow velocity of 22.4 cm s^{-1}, and an effective membrane area of 22.05 cm^2. All data for membrane performance parameters are presented as the mean ± SD from a minimum of three independent experiments.

Table S3. The adsorption capacity of the substrate for cyclen.

Membrane	Cyclen adsorption capacity (mg cm^{-2})
PES	0.0229±0.0002
PAN	0.0181±0.0003
PSF	0.0242±0.0012

Determination of cyclen adsorption capacity of substrates followed the procedure reported by Derrick S. Dlamini et al(*40*). An 81.00 cm^2 PES substrate was weighed and clamped into a polytetrafluoroethylene fixture. Subsequently, 20 mL of the amine solution, which was used for the preparation of the CON membrane, was injected, ensuring contact solely with the surface of the substrate. After 3 minutes, the solution was drained, and the excess amine solution was gently removed using a rubber roller. The specimen was then weighed after its immersion in the cycloalkene solution. The solution uptake was calculated by subtracting the initial dry weight from the final wet weight. The adsorption capacity of the substrates was calculated using the following equation: the product of the solution uptake and the cyclen concentration, divided by the area of the substrates. The data is presented as the mean ± SD of membrane performance parameters from at least three independent experiments.

Table S4. Ionic radius, hydrated radius, hydration energy, and separation performance of ions investigated in this study.

Type	Ions	Ionic Radius (Å)	Stokes Radius (Å)	Hydrated Radius (Å)	Hydration energy (kcal mol^{-1})	Salt used in the test	Rejection (%)	
							CON-T	CON-I
Cations	K^+	1.33	1.25	3.31	70.5	KCl	46.03	66.07
	Na^+	0.95	1.84	3.58	87.2	NaCl	54.82	77.89
	Li^+	0.6	2.38	3.82	113.5	LiCl	41.09	66.57
	Ba^{2+}	1.35	2.90	4.04	298.8	$BaCl_2$	97.32	98.27
	Ni^{2+}	0.70	2.92	4.04	473.2	$NiCl_2$	98.12	98.41
	Ca^{2+}	0.99	3.10	4.12	359.7	$CaCl_2$	96.91	98.31
	Co^{2+}	0.72	3.35	4.23	457.7	$CoCl_2$	97.38	98.20
	Mg^{2+}	0.65	3.47	4.23	437.4	$MgCl_2$	98.30	98.26
Anions	Cl^-	1.80	1.20	3.32	81.2	NaCl	54.82	77.89
	SO_4^{2-}	2.90	2.30	3.82	258.1	$NaSO_4$	98.25	98.90

Table S5. Water permeability, fitted ion permeabilities, and permeability ratios of literature membranes, commercial membranes, and CON membranes.

Membrane	Water permeance Pw (L m^{-2} h^{-1} bar^{-1})	S (Li^+/Mg^{2+})	P_{Li} (μm s^{-1})	P_{Mg} (μm s^{-1})	P_{Li}/P_{Mg}	Ref.
CON-T	22.2	59.7	8.44	0.044	191.79	This work
CON-T	22.2	66.7	13.54	0.046	294.28	
CON-T	22.2	58.8	16.16	0.055	294.31	
CON-T	22.2	82.6	34.75	0.047	733.03	
CON-T	22.2	54.3	20.33	0.065	312.22	
CON-I	9.8	175.3	2.49	0.008	319.56	
CON-I	9.8	195.2	4.55	0.011	417.22	
CON-I	9.8	161.9	3.34	0.011	315.08	
CON-I	9.8	326.7	6.29	0.008	767.56	
CON-I	9.8	249.9	7.27	0.012	626.47	
SIAIP-SDS	7.8	85.0	5.39	0.029	183.44	(*82*)
SIAIP-SDS	7.8	76.8	4.53	0.029	158.52	(*82*)
SIAIP-SDS	7.8	66.4	4.17	0.031	132.69	(*82*)
SIAIP-SDS	7.8	68.9	12.26	0.057	214.64	(*82*)
SIAIP-DDP	6.5	119.5	3.78	0.016	233.38	(*82*)
SIAIP-DDP	6.5	113.4	3.49	0.016	217.95	(*82*)
SIAIP-DDP	6.5	111.4	3.74	0.017	218.44	(*82*)
SIAIP-DDP	6.5	121.2	7.49	0.025	298.46	(*82*)
SIAIP	11.1	42.2	11.20	0.144	78.07	(*59*)
SIAIP	11.1	28.9	9.88	0.161	61.54	(*59*)
SIAIP	11.1	19.9	9.20	0.165	55.70	(*59*)
M-0 NF membrane	4.6	7.3	4.07	0.103	19.11	(*83*)
M-2 NF membrane	6.2	13.0	3.83	0.098	39.11	(*83*)
M-5 NF membrane	1.0	5.9	3.34	0.227	14.71	(*83*)
(PES-GO)/PEI/TMC	11.2	16.3	4.45	0.136	32.61	(*84*)
DAIB	15.5	16.6	12.10	0.223	54.25	(*50*)
DAIB	10.8	11.1	9.85	0.325	30.28	(*50*)
DAIB	3.9	5.7	6.13	0.464	13.21	(*50*)

PES-MWCNTS-COOK/TMC	11.5	58.7	4.98	0.040	123.17	(*85*)
Desal DL	6.1	5.1	32.23	1.210	26.59	(*86*)
Desal DL	6.1	2.8	39.12	5.140	7.61	(*86*)
PAN/DAPP/TMC	2.6	2.6	21.76	0.850	26.70	(*87*)
NF270	19.7	2.8	4.92	1.110	4.45	(*88*)
NF90	3.3	2.2	0.01	0.000	2.82	(*88*)
NF270	19.7	6.1	21.30	2.030	10.47	(*88*)
NF90	3.3	76.0	1.41	0.010	112.21	(*88*)
NF270	19.7	3.9	13.76	2.120	6.49	(*88*)
NF90	3.3	35.0	0.75	0.010	51.52	(*88*)
NF270	19.7	3.9	7.47	1.180	6.30	(*88*)
NF90	3.3	11.3	0.22	0.010	16.19	(*88*)
PEI/GQDs-NH2/TMC	11.9	26.7	3.00	0.070	42.84	(*89*)
Dual-skin layer NF	12.0	34.3	5.62	0.100	54.10	(*60*)
Dual-skin layer NF	12.0	34.6	6.69	0.110	58.94	(*60*)
Dual-skin layer NF	12.0	33.4	5.05	0.090	54.46	(*60*)
Dual-skin layer NF	12.0	28.3	3.27	0.070	46.39	(*60*)
Dual-skin layer NF	12.0	24.1	0.79	0.020	40.30	(*60*)
MBCN-0.02	5.6	25.0	1.47	0.040	37.98	(*90*)
MBCN-0.03	5.6	18.8	0.75	0.020	34.37	(*90*)
PES/CNC-COOH/PA	4.2	20.1	3.87	0.120	31.71	(*91*)
PES/CNC-COOH/PA	3.4	20.0	3.45	0.110	31.68	(*91*)
PEI-TMC	5.0	16.2	5.28	0.200	26.60	(*92*)
PIP-MWCNTs/PEI/TMC	14.0	16.4	8.45	0.320	26.40	(*93*)
NF-IL-2%	5.5	9.3	12.28	0.610	19.99	(*94*)
NF-IL-2%	5.5	8.1	13.02	0.680	19.01	(*94*)
NF-IL-2%	5.5	7.5	12.37	0.680	18.15	(*94*)
NF-IL-2%	5.5	6.2	14.43	0.830	17.42	(*94*)
PA-B2-E3	1.5	9.2	0.88	0.060	14.98	(*95*)
PES/(PIP-PHF)/TMC	6.7	12.9	9.09	0.390	23.23	(*96*)
MWCNTs-COOK	12.3	57.7	2.82	0.030	90.91	(*97*)

PES/CQDs-NH2-TMC	33.0	14.4	3.43	0.150	23.02	(*98*)
SPE-PEI600	10.0	12.0	13.02	0.600	21.64	(*99*)
SPE-PEI600	10.0	9.0	10.96	0.610	18.03	(*99*)
SPE-PEI600	10.0	7.0	3.33	0.210	16.07	(*99*)
TFN/PEI-3	30.6	36.5	13.49	0.230	60.52	(*100*)
Cu-MPD	16.2	8.0	8.44	0.660	12.81	(*101*)
PSS/PAH2.5(LbL)	7.9	279.0	0.88	0.002	420.24	(*102*)
PSS/PAH2.5(LbL)	7.9	370.3	0.85	0.002	568.27	(*102*)
PSS/PAH2.5(LbL)	7.9	381.6	0.82	0.001	584.43	(*102*)
PSS/PAH2.5(LbL)	7.9	250.2	1.61	0.004	393.27	(*102*)
PSS/PAH2.5(LbL)	7.9	249.2	2.09	0.005	445.17	(*102*)
PSS/PAH2.5(LbL)	7.9	153.2	2.68	0.009	287.67	(*102*)
PSS/PAH2.5-X(LbL)	6.2	262.3	1.42	0.004	406.83	(*102*)
PSS/PAH2.5-X(LbL)	6.2	255.2	1.11	0.003	383.03	(*102*)
PSS/PAH2.5-X(LbL)	6.2	330.2	0.99	0.002	494.95	(*102*)
PSS/PAH2.5-X(LbL)	6.2	196.2	2.05	0.006	325.78	(*102*)
PSS/PAH2.5-X(LbL)	6.2	176.7	2.08	0.006	330.46	(*102*)
PSS/PAH2.5-X(LbL)	6.2	126.0	2.38	0.008	289.78	(*102*)
PES/PSS/PAH(LbL)	18.4	71.4	4.54	0.041	109.95	(*103*)
PES/PSS/PAH(LbL)	18.4	65.5	4.10	0.041	99.77	(*103*)
PES/PSS/PAH(LbL)	18.4	74.3	4.08	0.036	112.57	(*103*)
PES/PSS/PAH(LbL)	18.4	61.0	5.23	0.050	103.78	(*103*)
PES/PSS/PAH(LbL)	18.4	45.8	6.45	0.073	88.50	(*103*)
NFX	2.1	16.9	7.02	0.150	46.37	(*45*)
NFX	2.1	6.6	9.96	0.420	23.74	(*45*)
NFX	2.1	15.4	8.14	0.190	42.92	(*45*)
NFX	2.1	13.1	7.23	0.180	39.88	(*45*)
NFX	2.1	13.9	6.19	0.140	45.42	(*45*)
NF90	3.3	20.8	1.64	0.050	32.90	(*45*)
NF90	3.3	19.2	2.14	0.070	31.34	(*45*)
NF90	3.3	18.5	2.05	0.070	29.83	(*45*)

NF90	3.3	21.7	1.59	0.040	35.60	(*45*)
NF90	3.3	22.7	1.17	0.030	38.32	(*45*)
NF270	19.7	2.3	65.69	8.860	7.41	(*45*)
NF270	19.7	1.8	127.47	18.350	6.95	(*45*)
NF270	19.7	2.1	80.87	10.930	7.40	(*45*)
NF270	19.7	2.3	64.30	8.580	7.49	(*45*)
NF270	19.7	2.3	66.43	7.800	8.51	(*45*)

We used open-source MATLAB code (https://github.com/ruoyuwang16/NATWATER-22-0394-Data-and-Codes) provided by Lin et al. to fit the ion permeability(*45*).

Table S6. Comparison of the Cl^-/SO_4^{2-} separation performances of the CON membranes, state-of-the-art nanofiltration membranes reported in the literature, and commercial membranes.

Membrane	Water permeance (L m^{-2} h^{-1} bar^{-1})	Rejection		S (Cl^-/SO_4^{2-})	Ref.
		Na_2SO_4 / SO_4^{2-}	NaCl / Cl^-		
PES-PIP/PPNs-TMC	37.4	98.4	18.3	5.1.1	(*104*)
BTC-PIP	8.7	99.1	83.3	18.6	(*105*)
TFN-AU4	30.8	97.5	20	31.9	(*106*)
SWCNT-TFC	40	96.5	13.4	24.7	(*107*)
(PEI-TMC)-COOH	12.5	92	17	8.4	(*108*)
PSF-G4D-1	26.4	99.2	41.1	73.6	(*109*)
PA50/CNC/PES	34	97	6.5	40.7	(*110*)
i-TFC-SG-6	22.1	97	33	22.3	(*111*)
Erythritol/TMC	7.5	95.9	78.7	5.2	(*112*)
IP-CFeM15	34.1	95	7.4	18.5	(*113*)
PE-XYL	4.28	94.3	75.1	4.4	(*114*)
PA/CLS (5)	53.6	94.3	27.3	12.8	(*115*)
TFNM with HZNCs	12.2	94.7	38.2	11.7	(*116*)
TFNM with ZNPs	14.5	92.3	36.8	8.2	(*116*)
Control TFC NF	10.5	92.1	32.7	8.5	(*116*)
CTN9	15.5	98.9	32.5	61.4	(*117*)
MT-0.75%	21.5	97.8	19	36.8	(*118*)
PA@DCA0.2%	8.6	99	13	87	(*119*)
MoS_2 TFN	7.9	97.9	65.1	16.6	(*120*)
PD/SWCNTs	32	95.9	22.5	18.9	(*121*)
TFN-mZIF2	14.3	93	11.5	12.6	(*122*)
TA-MoS22-TFN NFMs	17	98.5	28	48	(*123*)
PDA/PEG-TFN3	11.7	95	21.5	15.9	(*124*)
PA-W-CN	27.2	98.8	12	76	(*125*)
BHTTM/PIP (After oxidation)	13.2	99.5	30	140	(*126*)
PIP only @ hollow fiber	6.8	99.3	13.6	123	(*127*)
NFM-2	9.8	99.5	44	112	(*128*)
PEI-0.03 wt%	48	74	15.1	3.3	(*129*)

PD/ZIF-8/PES	53.5	95.2	10.9	18.6	(*130*)
NCM_0.025–0.05%	25.1	99.1	27.5	80.6	(*131*)
PA/GE20/PAN	33.7	98.1	14.3	45.1	(*132*)
TFN-HNTs	34.5	97.8	12.3	39.8	(*133*)
TFC-R	21.3	99.4	43.5	94.2	(*134*)
TFCn	19.7	95.2	17.2	20.6	(*135*)
PEI/TA-Psf NF with interlayer	10.8	99	48	52	(*136*)
PIP-TMC-QAEP	18.5	97.8	16.1	38.1	(*137*)
PIP-TMC	6.2	98.8	56.3	36.4	(*137*)
PIP 0.0175 wt%	52.8	96.4	17	23	(*138*)
PIP 0.015 wt%	62.9	93.5	10.5	13.8	(*138*)
PIP-0.8-60	22	99	23.4	76.6	(*139*)
PIP-0.3-60	34.7	95.5	12.2	19.5	(*139*)
M-Control	4.5	98.1	27.3	38.2	(*139*)
M-U1-A	9.7	95.7	25.1	17.4	(*139*)
M-U4-O	7.9	99.6	31.7	170.7	(*139*)
PA/M-50 (PIP 0.05/TMC 0.05)	26.2	97.7	17	36	(*140*)
PES-COFs scaffold/PIP-TMC	31.1	95	11.9	17.6	(*141*)
Alginate NFMs	13.1	97.6	12.7	36.4	(*142*)
PA@A	5.6	92	23	9.6	(*126*)
PA@W-7	9.4	98.5	43	38	(*126*)
PA@W-0	27.5	98.5	52.5	31.6	(*126*)
PA@W-14	26.5	98.4	28.3	44.8	(*126*)
PA20/PAN TFNC	25.8	99.1	26.3	82	(*143*)
NF-PIP/TMC	5.7	95.8	49.5	12	(*144*)
NF-0.1%-CB-1	15.5	94.9	18.3	16	(*144*)
TFC-Control	4.9	97.2	40.5	21.2	(*145*)
TFC-SDS	7.5	92.3	47	6.9	(*145*)
TFC-PDA/PEI	15	97.5	42.5	23	(*146*)
TFC-TA/PEI	16.7	97.8	43.6	25.6	(*146*)
TFC-ZIF-8/PEI	19.9	98.2	45.2	30.4	(*146*)

TFC-PEG	15.5	97.4	52.8	18.1	(*146*)
TFC-PVP	18.4	98.2	53.6	25.8	(*146*)
TFC-PVA	24.6	98.4	54.2	28.6	(*146*)
PSA-PSF	0.78	91.7	59.4	4.8	(*147*)
PSA/SPEEK-PSF	1.9	99.4	88.5	19.2	(*147*)
SPEEK-PSF	4.6	94.8	64.5	6.8	(*147*)
TPT-TMC/PSf TFC	9.3	98.6	40.5	42.5	(*148*)
PIP-TMC/PSf TFC	8.2	97.6	54.2	19.1	(*148*)
PVC-NF2	7	98	30	35	(*149*)
PA/PES/PANI-0.2	15.7	95	33	13.4	(*150*)
TFN-4H	19.4	95.2	47.4	10.9	(*151*)
TFN-4S	13.4	93.5	41.2	9	(*151*)
TFC_0	6.8	91.1	25	8.4	(*152*)
TFC50	9.8	97.8	21.9	35.5	(*152*)
TFC90	20.2	81.3	17.4	4.4	(*152*)
PA-SNW-1/PES	19.2	83.5	15.3	5.1	(*153*)
PIP/dopamine-0	17.5	69.7	15	2.8	(*154*)
PIP/dopamine-1.0	13.5	94.8	21	15.2	(*154*)
PIP/dopamine-2.5	10.8	96.8	23.7	23.8	(*154*)
PIP/GLA-TMC TFNC	28.5	96.4	14.1	7	(*155*)
PA-ATP (5) /PES	22.9	92	14.7	10.7	(*156*)
i-TFC0.025	30.7	83.7	~12	5.4	(*157*)
Nylon-2.5 TFC	42.4	97	7.7	30.8	(*158*)
NF 40	2.15	95	45	11	(*104*)
Nitto-Denko NTR-7450	10.9	92	53	5.9	(*104*)
Toray UTC20	10.2	93	55	6.4	(*104*)
NTR-7250	6.3	99	55	45	(*104*)
NS-300	4.9	97.8	70	13.6	(*104*)
Dow-Filmtec NF 270	13.2	99.2	49.5	63.1	(*135*)
Dow-Filmtec NF 90	6.5	98.2	64.4	19.8	(*135*)
Dow-Filmtec NF 70	7.2	97	70	10	(*159*)

GE-Osmonics DL	10	96	40	15	(*159*)
GE-Osmonics HL	6.9	97	33	23.3	(*159*)
Synder NFX	2.4	99	40	60	(*159*)
Synder NFW	5.4	97	20	26.7	(*159*)
Dow-Filmtec NF 270	11.6	94	51	14.8	(*160*)
Dow-Filmtec NF 270	11.3	98.2	59.2	36.9	(*161*)
Dow-Filmtec NF 270	15.2	96.1	25.9	75	(*162*)
Dow-Filmtec NF 270	21.5	95	71	7.9	(*163*)
N-TFN	41.7	98.7	34.4	50	(*19*)
PIP only	10.91	98.4	30.2	43.6	(*164*)
PIP only	10.91	96.1	25.5	24.5	(*164*)
H40/PIP	16.25	99.3	15.9	120	(*164*)
H40/PIP	16.25	98.5	10,6	59.6	(*164*)
A-CPTC-PIP	13.34	98.9	15.5	76.8	(*165*)
A-CPTC-PIP	13.34	98.9	-8.24	98.4	(*165*)
DK	6.84	98.5	46.6	35.6	(*165*)
DK	6.84	98.8	21.3	65.6	(*165*)
DL	4.73	98.6	47.95	37.2	(*165*)
DL	4.73	98.9	20.54	72.2	(*165*)
NF270	10.74	98.7	50.84	37.8	(*165*)
NF270	10.74	98.9	23.63	69.4	(*165*)
XC-N	8.97	98.6	51.4	34.7	(*165*)
XC-N	8.97	98.7	24.7	57.9	(*165*)
Pre-diffusion IP	6.4	99.7	26.2	246	(*166*)
PA/MPC	8.5	N.A.	N.A.	93	(*167*)
e-IP3	44.7	94.7	-8.2	20.4	(*168*)
e-IP3	44.7	94.8	-9.9	21.1	(*168*)
NF0	12.0	97.2	40.6	40.6	(*169*)
NF1	14.8	97.2	35.3	23.1	(*169*)
NF2	16.4	97.3	32	25.2	(*169*)
NFMs@SMPS12h	23.6	95.1	-15.2	23.6	(*170*)

G5-TAC2/PIP	10.35	N.A.	N.A.	65.8	(*171*)
SCOF/PA	12.6	99.6	28.6	178.5	(*172*)
TFC-0 NFMS	7.7	90.1	17.4	8.4	(*173*)
TFC-2 NFMS	20.9	93.2	18.8	85.5	(*173*)
NF110	8.4	N.A.	N.A.	40.8	(*174*)
PIP/COF-A/PES	16.3	N.A.	N.A.	74	(*175*)
CON-T	22.2	99.00	27.55	72.5	This work
CON-T	22.2	99.22	12.06	112.9	
CON-T	22.2	99.22	9.75	118.1	
CON-I	9.8	99.78	61.98	164.2	
CON-I	9.8	99.83	44.2	326.7	
CON-I	9.8	99.83	37.69	376.9	

Table S7. Comparison of the Li^+ purity and Li^+ recovery of the CON membranes, and recent literature containing Li^+ purity and Li^+ recovery data.

Membrane	Water permeance (L m^{-2} h^{-1} bar^{-1})	Water recovery	Li^+ recovery	Li^+ purity	Ref.
XN45	13.3	0.10	0.10	0.62	(*8*)
XN45	13.3	0.20	0.20	0.62	(*8*)
XN45	13.3	0.30	0.31	0.61	(*8*)
XN45	13.3	0.40	0.42	0.60	(*8*)
XN45	13.3	0.50	0.53	0.59	(*8*)
XN45	13.3	0.55	0.59	0.59	(*8*)
DK	4.4	0.02	0.04	0.61	(*176*)
DK	4.4	0.04	0.08	0.68	(*176*)
DK	4.4	0.06	0.12	0.66	(*176*)
DK	4.4	0.07	0.13	0.65	(*176*)
DK	4.4	0.08	0.14	0.68	(*176*)
DK	4.4	0.33	0.53	0.77	(*176*)
DK	4.4	0.50	0.79	0.71	(*176*)
DK	4.4	0.66	0.97	0.57	(*176*)
DK	4.4	0.75	0.99	0.44	(*176*)
DK[1]	7.7	0.50	0.83	0.49	(*177*)
DL	5.7	N.A.	0.55	0.15	(*178*, *179*)
NF270	10.8	0.33	0.51	0.57	(*180*)
NF270	10.8	0.50	0.73	0.47	(*180*)
NF270	10.8	0.67	0.83	0.32	(*180*)
NF270	10.8	0.75	0.92	0.27	(*180*)
$TFC_{1.0}$	10.7	0.50	0.31	0.30	(*177*)
$PIP_{0.8}/PEI_{0.2}$	11.5	0.50	0.31	0.52	(*177*)
$PIP_{0.6}/PEI_{0.4}$	12.1	0.50	0.30	0.62	(*177*)
$PIP_{0.4}/PEI_{0.6}$	11.8	0.50	0.29	0.53	(*177*)
$PIP_{0.2}/PEI_{0.8}$	11.9	0.50	0.31	0.39	(*177*)
$PIP_{0}/PEI_{1.0}$	10.8	0.50	0.33	0.33	(*177*)
$TFC_{0.35}$	26.5	0.50	0.67	0.07	(*177*)

$TFC_{0.35}$-PEI_{600}	12.9	0.50	0.59	0.42	(*177*)
$TFC_{0.35}$-PEI_{3000}	13.1	0.50	0.47	0.63	(*177*)
$TFC_{0.35}$-PEI_{10000}	14.1	0.50	0.60	0.41	(*177*)
DK*	5.1	0.13	0.13	0.78	(*178*, *181*)
DK*	5.1	0.17	0.21	0.78	(*178*, *181*)
DK*	5.1	0.25	0.38	0.79	(*178*, *181*)
DK*	5.1	0.33	0.52	0.80	(*178*, *181*)
DK*	5.1	0.42	0.66	0.80	(*178*, *181*)
DK*	5.1	0.50	0.79	0.69	(*178*, *181*)
DK*	5.1	0.58	0.93	0.61	(*178*, *181*)
CON-T	22.2	0.8075	0.974	0.993	This work

* Li^+ purity calculations only take in to account Li^+ and Mg^{2+}, where data for other cations are not reported.

Table S8. Parameters of two/three-pass nanofiltration with brine recirculation for CON-T and DK membrane.

Membrane	Pass	WR	Pressure (bar)	Flow rate (m^3 h^{-1})	$C_{f,Li}$ (mM)	$C_{f,Mg}$ (mM)	Feed MLR	$C_{p,Li}$ (mM)	$C_{p,Mg}$ (mM)	$C_{c,Li}$ (mM)	$C_{c,Mg}$ (mM)	Li^+ purity	Li^+ recovery (local)	Li^+ recovery (cumulative)
CON-T	1	0.95	23.01	11.66	6.159	25.471	14.487	6.262	2.68	2.016	459.430	0.400	0.966	
	2	0.85	6.69	11.08	6.262	2.683	1.501	4.635	0.009	15.478	17.834	***0.993***	0.629	***0.974***
DK	1	0.95	19.37	11.66	4.301	31.186	25.402	4.485	9.621	0.802	440.860	0.117	0.991	
	2	0.85	6.17	11.08	4.485	9.621	7.515	4.846	1.088	2.438	57.939	***0.560***		***0.990***
DK	1	0.95	19.92	11.95	4.403	31.609	25.147	4.591	9.738	0.841	447.050	0.119	0.990	
	2	0.85	6.99	13.01	5.097	9.186	6.313	5.406	0.835	3.343	56.549	0.649	0.902	
	3	0.85	4.80	11.06	5.406	0.835	0.541	4.849	0.026	8.559	5.409	***0.981***	0.762	***0.989***

Table S9. Properties of PES substrate.

Project	value
Pore size* (nm)	13.4±3.7
Surface porosity* (%)	10.7±1.4
Body porosity† (%)	29.9±1.3
Water permeance‡ (L m^{-2} h^{-1} bar^{-1})	171.2±8.1
Water contact angle (°)	78.5±1.9

* Pore size and surface porosity were obtained through statistical analysis of SEM images.

† Determination of body porosity follows the procedure reported by Derrick S. Dlamini et al(*40*). An 81.00 cm² PES substrate was weighed and clamped into a polytetrafluoroethylene (PTFE) fixture. Subsequently, 20 mL of the amine solution, which was used for the preparation of the CON membrane, was injected, ensuring contact solely with the surface of the substrate. After 3 minutes, the solution was drained, and the excess amine solution was gently removed using a rubber roller. The specimen was then weighed after its immersion in the cycloalkene solution. The solution uptake was calculated by subtracting the initial dry weight from the final wet weight. The bulk porosity of the support membrane was defined as the ratio of the volume (ratio of mass to density) of the adsorbed amine solution to the volume of the substrate. The data is presented as the mean ± SD of membrane performance parameters from at least three independent experiments.

‡ Water permeance was measured through cross-flow device. The test temperature was 25 ± 1 °C, the cross-flow velocity was 22.4 cm s^{-1}, the pressure was 6.0 bar and the effective membrane area was 22.05 cm^2. The data is presented as the mean ± SD of membrane performance parameters from at least three independent experiments.

Table S10. Simulation parameters for 21-step equilibrium.

Step	Temperature (K)	Pressure (bar)	Ensemble	Simulation time (ps)
1	600	/	NVT	50
2	300	/	NVT	50
3	300	1000	NPT	50
4	600	/	NVT	50
5	300	/	NVT	100
6	300	3000	NPT	50
7	600	/	NVT	50
8	300	/	NVT	100
9	300	5000	NPT	50
10	600	/	NVT	50
11	300	/	NVT	100
12	300	2500	NPT	50
13	600	/	NVT	5
14	300	/	NVT	10
15	300	500	NPT	5
16	600	/	NVT	5
17	300	/	NVT	10
18	300	50	NPT	5
19	600	/	NVT	5
20	300	/	NVT	10
21	300	1	NPT	800

Supplementary Movies

Movie S1

Trajectory file for evaluating the PMF of Li^{+} through the CON-T membrane.

Movie S2

Trajectory file for evaluating the PMF of Mg^{2+} through the CON-T membrane.

REFERENCES

1. P. Zuo, C. Ye, Z. Jiao, J. Luo, J. Fang, U. S. Schubert, N. B. McKeown, T. L. Liu, Z. Yang, T. Xu, Near-frictionless ion transport within triazine framework membranes. *Nature* **617**, 299–305 (2023).

2. A. Iddya, P. Zarzycki, R. Kingsbury, C. M. Khor, S. Ma, J. Wang, I. Wheeldon, Z. J. Ren, E. M. V. Hoek, D. Jassby, A reverse-selective ion exchange membrane for the selective transport of phosphates via an outer-sphere complexation–diffusion pathway. *Nat. Nanotechnol.* **17**, 1222–1228 (2022).

3. J. Yang, B. Tu, G. Zhang, P. Liu, K. Hu, J. Wang, Z. Yan, Z. Huang, M. Fang, J. Hou, Q. Fang, X. Qiu, L. Li, Z. Tang, Advancing osmotic power generation by covalent organic framework monolayer. *Nat. Nanotechnol.* **17**, 622–628 (2022).

4. J. Lu, H. Zhang, J. Hou, X. Li, X. Hu, Y. Hu, C. D. Easton, Q. Li, C. Sun, A. W. Thornton, M. R. Hill, X. Zhang, G. Jiang, J. Z. Liu, A. J. Hill, B. D. Freeman, L. Jiang, H. Wang, Efficient metal ion sieving in rectifying subnanochannels enabled by metal–organic frameworks. *Nat. Mater.* **19**, 767–774 (2020).

5. R. Epsztein, R. M. DuChanois, C. L. Ritt, A. Noy, M. Elimelech, Towards single-species selectivity of membranes with subnanometre pores. *Nat. Nanotechnol.* **15**, 426–436 (2020).

6. Y. Zhang, K. Zhou, S. Su, J. Gao, J. Liu, L. Jiang, Congener-welded crystalline carbon nitride membrane for robust and highly selective Li/Mg separation. *Sci. Adv.* **10**, eadm9620 (2024).

7. Z. Li, I.-C. Chen, L. Cao, X. Liu, K.-W. Huang, Z. Lai, Lithium extraction from brine through a decoupled and membrane-free electrochemical cell design. *Science* **385**, 1438–1444 (2024).

8. M. Yong, M. Tang, L. Sun, F. Xiong, L. Xie, G. Zeng, X. Ren, K. Wang, Y. Cheng, Z. Li, E. Li, X. Zhang, H. Wang, Sustainable lithium extraction and magnesium hydroxide co-production from salt-lake brines. *Nat. Sustain.* **7**, 1662–1671 (2024).

9. C. Lu, C. Hu, Z. Chen, P. Wang, F. Feng, G. He, F. Wang, Y. Zhang, J. Z. Liu, X. Zhang, J. Qu, Dehydration-enhanced ion-pore interactions dominate anion transport and selectivity in nanochannels. *Sci. Adv.* **9**, eadf8412 (2023).

10. Q. Wang, Y. Wang, B.-Z. Chen, T.-D. Lu, H.-L. Wu, Y.-Q. Fan, W. Xing, S.-P. Sun, Designing high-performance nanofiltration membranes for high-salinity separation of sulfate and chloride in the chlor-alkali process. *Ind. Eng. Chem. Res.* **58**, 12280–12290 (2019).

11. R. M. DuChanois, C. J. Porter, C. Violet, R. Verduzco, M. Elimelech, Membrane materials for selective ion separations at the water–energy nexus. *Adv. Mater.* **33**, e2101312 (2021).

12. X. Lu, M. Elimelech, Fabrication of desalination membranes by interfacial polymerization: History, current efforts, and future directions. *Chem. Soc. Rev.* **50**, 6290–6307 (2021).

13. P. Sarkar, C. Wu, Z. Yang, C. Y. Tang, Empowering ultrathin polyamide membranes at the water–energy nexus: Strategies, limitations, and future perspectives. *Chem. Soc. Rev.* **53**, 4374–4399 (2024).

14. H. B. Park, J. Kamcev, L. M. Robeson, M. Elimelech, B. D. Freeman, Maximizing the right stuff: The trade-off between membrane permeability and selectivity. *Science* **356**, eaab0530 (2017).

15. Z. Yang, H. Guo, C. Y. Tang, The upper bound of thin-film composite (TFC) polyamide membranes for desalination. *J. Membr. Sci.* **590**, 117297 (2019).

16. Z. Tan, S. Chen, X. Peng, L. Zhang, C. Gao, Polyamide membranes with nanoscale Turing structures for water purification. *Science* **360**, 518–521 (2018).

17. L. Shen, R. Cheng, M. Yi, W.-S. Hung, S. Japip, L. Tian, X. Zhang, S. Jiang, S. Li, Y. Wang, Polyamide-based membranes with structural homogeneity for ultrafast molecular sieving. *Nat. Commun.* **13**, 500 (2022).

18. C. Zhao, Y. Zhang, Y. Jia, B. Li, W. Tang, C. Shang, R. Mo, P. Li, S. Liu, S. Zhang, Polyamide membranes with nanoscale ordered structures for fast permeation and highly selective ion-ion separation. *Nat. Commun.* **14**, 1112 (2023).

19. S. Han, J. Zhu, A. A. Uliana, D. Li, Y. Zhang, L. Zhang, Y. Wang, T. He, M. Elimelech, Microporous organic nanotube assisted design of high performance nanofiltration membranes. *Nat. Commun.* **13**, 7954 (2022).

20. Y. Liang, Y. Zhu, C. Liu, K.-R. Lee, W.-S. Hung, Z. Wang, Y. Li, M. Elimelech, J. Jin, S. Lin, Polyamide nanofiltration membrane with highly uniform sub-nanometre pores for sub-1 Å precision separation. *Nat. Commun.* **11**, 2015 (2020).

21. W. Meng, S. Chen, M. Wu, F. Gao, Y. Hou, X. Zhan, W. Hu, L. Liang, Q. Zhang, Dehydration-enhanced ion recognition of triazine covalent organic frameworks for high-resolution Li^{+} /Mg^{2+} separation. *Angew. Chem. Int. Ed. Engl.* **64**, e202422423 (2025).

22. W. Meng, S. Chen, Z. Guo, F. Gao, J. Wang, J. Lu, Y. Hou, Q. He, X. Zhan, M. Qiu, Q. Zhang, Three-dimensional cationic covalent organic framework membranes for rapid and selective lithium extraction from saline water. *Nat. Water* **3**, 191–200 (2025).

23. L. Cao, I.-C. Chen, C. Chen, D. B. Shinde, X. Liu, Z. Li, Z. Zhou, Y. Zhang, Y. Han, Z. Lai, Giant osmotic energy conversion through vertical-aligned ion-permselective nanochannels in covalent organic framework membranes. *J. Am. Chem. Soc.* **144**, 12400–12409 (2022).

24. M. Wang, P. Zhang, X. Liang, J. Zhao, Y. Liu, Y. Cao, H. Wang, Y. Chen, Z. Zhang, F. Pan, Z. Zhang, Z. Jiang, Ultrafast seawater desalination with covalent organic framework membranes. *Nat. Sustain.* **5**, 518–526 (2022).

25. Y. Kang, Y. Wang, H. Zhang, Z. Wang, X. Zhang, H. Wang, Functionalized 2D membranes for separations at the 1-nm scale. *Chem. Soc. Rev.* **53**, 7939–7959 (2024).

26. J. Hong, M. Liu, Y. Liu, S. Shang, X. Wang, C. Du, W. Gao, C. Hua, H. Xu, Z. You, Y. Liu, J. Chen, Solid-liquid interfacial engineered large-area two-dimensional covalent organic framework films. *Angew. Chem. Int. Ed. Engl.* **63**, e202317876 (2024).

27. J. R. McCutcheon, M. S. Mauter, Fixing the desalination membrane pipeline. *Science* **380**, 242–244 (2023).

28. S. Wu, L. E. Peng, Z. Yang, P. Sarkar, M. Barboiu, C. Y. Tang, A. G. Fane, Next-generation desalination membranes empowered by novel materials: Where are we now? *Nano Micro Lett.* **17**, 91 (2025).

29. D. Beaudoin, T. Maris, J. D. Wuest, Constructing monocrystalline covalent organic networks by polymerization. *Nat. Chem.* **5**, 830–834 (2013).

30. B. Jiang, J. Zhang, K. Yu, Z. Jia, H. Long, N. He, Y. Zhang, Y. Zou, Z. Han, Y. Li, L. Ma, Dynamic cleavage-remodeling of covalent organic networks into multidimensional superstructures. *Adv. Mater.* **36**, e2404446 (2024).

31. J. Liu, S. Wang, T. Huang, P. Manchanda, E. Abou-Hamad, S. P. Nunes, Smart covalent organic networks (CONs) with "on-off-on" light-switchable pores for molecular separation. *Sci. Adv.* **6**, eabb3188 (2020).

32. Z. Wang, K. Nakagawa, K. Guan, M. Hu, Z. Mai, W. Fu, Q. Shen, Y. Okamoto, A. Matsuoka, E. Kamio, T. Yoshioka, H. Matsuyama, Self-aggregation control of porphyrin for enhanced selective covalent organic network membranes. *Small* **21**, e2407986 (2025).

33. Z. Ou, B. Liang, Z. Liang, F. Tan, X. Dong, L. Gong, P. Zhao, H. Wang, Y. Zou, Y. Xia, X. Chen, W. Liu, H. Qi, U. Kaiser, Z. Zheng, Oriented growth of thin films of covalent organic frameworks with large single-crystalline domains on the water surface. *J. Am. Chem. Soc.* **144**, 3233–3241 (2022).

34. Z. Jiang, R. Dong, A. M. Evans, N. Biere, M. A. Ebrahim, S. Li, D. Anselmetti, W. R. Dichtel, A. G. Livingston, Aligned macrocycle pores in ultrathin films for accurate molecular sieving. *Nature* **609**, 58–64 (2022).

35. A. Yao, J. Du, Q. Sun, L. Liu, Z. Song, W. He, J. Liu, Flexible covalent organic network with ordered honeycomb nanoarchitecture for molecular separations. *ACS Nano* **17**, 22916–22927 (2023).

36. C. Y. Tang, Y.-N. Kwon, J. O. Leckie, Effect of membrane chemistry and coating layer on physiochemical properties of thin film composite polyamide RO and NF membranes. *Desalination* **242**, 149–167 (2009).

37. R. Dai, H. Zhou, T. Wang, Z. Qiu, L. Long, S. Lin, C. Y. Tang, Z. Wang, Nanovehicle-assisted monomer shuttling enables highly permeable and selective nanofiltration membranes for water purification. *Nat. Water* **1**, 281–290 (2023).

38. C. Jin, W. Zhang, N. Tian, B. Wu, M. Yin, Q. An, Fabrication of coffee-ring nanostructured membranes for organic solvent nanofiltration. *Angew. Chem. Int. Ed. Engl.* **63**, e202405891 (2024).

39. S. Shao, F. Zeng, L. Long, X. Zhu, L. E. Peng, F. Wang, Z. Yang, C. Y. Tang, Nanofiltration membranes with crumpled polyamide films: A critical review on mechanisms, performances, and environmental applications. *Environ. Sci. Technol.* **56**, 12811–12827 (2022).

40. D. S. Dlamini, J. A. Quezada-Renteria, J. Wu, M. Xiao, M. Anderson, R. B. Kaner, A. Edalat, N. Voutchkov, A. Al-Ahmoudi, E. M. V. Hoek, On the role of the porous support membrane in seawater reverse osmosis membrane synthesis, properties and performance. *J. Membr. Sci.* **708**, 123032 (2024).

41. X.-G. Jin, H. Y. Lim, Q. Wang, R. Jia, X.-H. Ma, Z.-L. Xu, C. Y. Tang, Enhanced thin-film composite nanofiltration membranes via substrate pore structure engineering: Performance and mechanistic insights. *Environ. Sci. Technol.* **59**, 15538–15546 (2025).

42. R. M. DuChanois, M. Heiranian, J. Yang, C. J. Porter, Q. Li, X. Zhang, R. Verduzco, M. Elimelech, Designing polymeric membranes with coordination chemistry for high-precision ion separations. *Sci. Adv.* **8**, eabm9436 (2022).

43. N. Gan, Y. Lin, B. Wu, Y. Qiu, H. Sun, J. Su, J. Yu, Q. Lin, H. Matsuyama, Supramolecular-coordinated nanofiltration membranes with quaternary-ammonium Cyclen for efficient lithium extraction from high magnesium/lithium ratio brine. *Water Res.* **268**, 122703 (2025).

44. T. Li, X. Zhang, Y. Zhang, J. Wang, Z. Wang, S. Zhao, Nanofiltration membrane comprising structural regulator Cyclen for efficient Li^{+}/Mg^{2+} separation. *Desalination* **556**, 116575 (2023).

45. R. Wang, R. He, T. He, M. Elimelech, S. Lin, Performance metrics for nanofiltration-based selective separation for resource extraction and recovery. *Nat. Water* **1**, 291–300 (2023).

46. B. Yuan, Y. Zhang, P. Qi, D. Yang, P. Hu, S. Zhao, K. Zhang, X. Zhang, M. You, J. Cui, J. Jiang, X. Lou, Q. J. Niu, Self-assembled dendrimer polyamide nanofilms with enhanced effective pore area for ion separation. *Nat. Commun.* **15**, 471 (2024).

47. A. Razmjou, M. Asadnia, E. Hosseini, A. Habibnejad Korayem, V. Chen, Design principles of ion selective nanostructured membranes for the extraction of lithium ions. *Nat. Commun.* **10**, 5793 (2019).

48. X. Zhai, S. Lin, X. Li, Z. Wang, The hidden role of the dielectric effect in nanofiltration: A novel perspective to unravel new ion separation mechanisms. *Environ. Sci. Technol.* **58**, 15874–15884 (2024).

49. W. Zhang, S. Zhao, H. Li, C. Lai, S. Zhang, W. Wen, C. Y. Tang, F. Meng, Lignin alkali regulated interfacial polymerization towards ultra-selective and highly permeable nanofiltration membrane. *Nat. Commun.* **16**, 371 (2025).

50. H. Peng, Q. Zhao, A nano-heterogeneous membrane for efficient separation of lithium from high magnesium/lithium ratio brine. *Adv. Funct. Mater.* **31**, 2009430 (2021).

51. J. Li, H. Peng, K. Liu, Q. Zhao, Polyester nanofiltration membranes for efficient cations separation. *Adv. Mater.* **36**, 2309406 (2024).

52. M. Heiranian, R. M. DuChanois, C. L. Ritt, C. Violet, M. Elimelech, Molecular simulations to elucidate transport phenomena in polymeric membranes. *Environ. Sci. Technol.* **56**, 3313–3323 (2022).

53. X. Zhou, Z. Wang, R. Epsztein, C. Zhan, W. Li, J. D. Fortner, T. A. Pham, J.-H. Kim, M. Elimelech, Intrapore energy barriers govern ion transport and selectivity of desalination membranes. *Sci. Adv.* **6**, eabd9045 (2020).

54. E. R. Nightingale, Phenomenological theory of ion solvation. Effective radii of hydrated ions. *J. Phys. Chem.* **63**, 1381–1387 (1959).

55. Y. Marcus, Thermodynamics of solvation of ions. Part 6.—The standard partial molar volumes of aqueous ions at 298.15 K. *J. Chem. Soc. Faraday Trans.* **89**, 713–718 (1993).

56. B. Wu, N. Gan, Y. Lin, Y. Zhang, J. Zhang, Y. Qiu, X. Cao, J. Yu, H. Matsuyama, Ion-selective transport promotion enabled by angstrom-scale nanochannels in dendrimer-assembled polyamide nanofilm for efficient electrodialysis. *Nano Lett.* **24**, 8650–8657 (2024).

57. H. Sun, N. Wang, Y. Xu, F. Wang, J. Lu, H. Wang, Q.-F. An, Aromatic-aliphatic hydrocarbon separation with oriented monolayer polyhedral membrane. *Science* **386**, 1037–1042 (2024).

58. Y. Bai, B. Liu, J. Li, M. Li, Z. Yao, L. Dong, D. Rao, P. Zhang, X. Cao, L. F. Villalobos, C. Zhang, Q.-F. An, M. Elimelech, Microstructure optimization of bioderived polyester nanofilms for antibiotic desalination via nanofiltration. *Sci. Adv.* **9**, eadg6134 (2023).

59. R. Jia, X.-G. Jin, Z.-L. Xu, L.-K. Wu, Y.-H. Tong, H.-X. Li, H.-H. Ping, X.-H. Ma, S.-J. Xu, Surfactant-interlayer assisted interfacial polymerization for constructing Janus nanofiltration membranes: Enhanced Li^+/Mg^{2+} separation efficiency. *J. Membr. Sci.* **712**, 123235 (2024).

60. Z. Yang, W. Fang, Z. Wang, R. Zhang, Y. Zhu, J. Jin, Dual-skin layer nanofiltration membranes for highly selective Li^+/Mg^{2+} separation. *J. Membr. Sci.* **620**, 118862 (2021).

61. F. Yang, M. Yong, Z. Li, Z. Yang, X. Zhang, Breaking the trade-off between lithium purity and lithium recovery: A comprehensive mathematical modeling based on membrane structure-property-performance relationships. *Water Res.* **281**, 123678 (2025).

62. R. Wang, R. Alghanayem, S. Lin, Multipass nanofiltration for lithium separation with high selectivity and recovery. *Environ. Sci. Technol.* **57**, 14464–14471 (2023).

63. Y. Su, H. Peng, X. Liu, J. Li, Q. Zhao, High performance, pH-resistant membranes for efficient lithium recovery from spent batteries. *Nat. Commun.* **15**, 10295 (2024).

64. L. Liu, S. Lin, X. Xu, Y. Wan, W. Song, J. Luo, Preference of negatively charged membranes in magnesium and lithium separation by nanofiltration. *Nat. Commun.* **16**, 5731 (2025).

65. J. Kim, J. F. Kim, Z. Jiang, A. G. Livingston, Advancing membrane technology in organic liquids towards a sustainable future. *Nat. Sustain.* **8**, 594–605 (2025).

66. X.-G. Jin, X. Tang, T.-X. Ren, J. Wang, P. Zheng, X.-H. Ma, Z.-L. Xu, Ionic liquids tailored ultra-permeable antifouling nanofiltration membranes for water purification. *J. Membr. Sci.* **696**, 122536 (2024).

67. L. J. Abbott, K. E. Hart, C. M. Colina, Polymatic: A generalized simulated polymerization algorithm for amorphous polymers. *Theor. Chem. Acc.* **132**, 1334 (2013).

68. T. F. Willems, C. H. Rycroft, M. Kazi, J. C. Meza, M. Haranczyk, Algorithms and tools for high-throughput geometry-based analysis of crystalline porous materials. *Microporous Mesoporous Mater.* **149**, 134–141 (2012).

69. J. S. Hub, B. L. De Groot, D. Van Der Spoel, g_wham—A free weighted histogram analysis implementation including robust error and autocorrelation estimates. *J. Chem. Theory Comput.* **6**, 3713–3720 (2010).

70. M. J. Abraham, T. Murtola, R. Schulz, S. Páll, J. C. Smith, B. Hess, E. Lindahl, GROMACS: High performance molecular simulations through multi-level parallelism from laptops to supercomputers. *SoftwareX* **1-2**, 19–25 (2015).

71. S. Nosé, A unified formulation of the constant temperature molecular dynamics methods. *J. Chem. Phys.* **81**, 511–519 (1984).

72. U. Essmann, L. Perera, M. L. Berkowitz, T. Darden, H. Lee, L. G. Pedersen, A smooth particle mesh Ewald method. *J. Chem. Phys.* **103**, 8577–8593 (1995).

73. B. Hess, H. Bekker, H. J. C. Berendsen, J. G. E. M. Fraaije, LINCS: A linear constraint solver for molecular simulations. *J. Comput. Chem.* **18**, 1463–1472 (1997).

74. R. Wang, S. Lin, Pore model for nanofiltration: History, theoretical framework, key predictions, limitations, and prospects. *J. Membr. Sci.* **620**, 118809 (2021).

75. W. R. Bowen, J. S. Welfoot, Modelling the performance of membrane nanofiltration—Critical assessment and model development. *Chem. Eng. Sci.* **57**, 1121–1137 (2002).

76. V. Geraldes, A. M. Brites Alves, Computer program for simulation of mass transport in nanofiltration membranes. *J. Membr. Sci.* **321**, 172–182 (2008).

77. E. M. V. Hoek, J. Allred, T. Knoell, B.-H. Jeong, Modeling the effects of fouling on full-scale reverse osmosis processes. *J. Membr. Sci.* **314**, 33–49 (2008).

78. A. R. Costa, M. N. de Pinho, Performance and cost estimation of nanofiltration for surface water treatment in drinking water production. *Desalination* **196**, 55–65 (2006).

79. D. Rehman, J. H. Lienhard, Global optimization for accurate and efficient parameter estimation in nanofiltration. *J. Membr. Sci. Lett.* **2**, 100034 (2022).

80. Q. Peng, R. Wang, Z. Zhao, S. Lin, Y. Liu, D. Dong, Z. Wang, Y. He, Y. Zhu, J. Jin, L. Jiang, Extreme Li-Mg selectivity via precise ion size differentiation of polyamide membrane. *Nat. Commun.* **15**, 2505 (2024).

81. W. Liu, J. L. Livingston, L. Wang, Z. Wang, M. Del Cerro, S. A. Younssi, R. Epsztein, M. Elimelech, S. Lin, Pressure-driven membrane desalination. *Nat. Rev. Methods Primers* **4**, 10 (2024).

82. R. Jia, H.-X. Li, Z.-L. Xu, X.-G. Jin, L.-K. Wu, H.-H. Ping, X.-H. Ma, S.-J. Xu, Surfactants intervened construction of NF membranes for lithium extraction in high Mg^{2+}/Li^{+} ratio and high concentration environments. *J. Membr. Sci.* **726**, 124048 (2025).

83. H. Wu, H. Zhao, Y. Lin, X. Liu, L. Wang, H. Yao, Y. Tang, L. Yu, H. Wang, X. Wang, Positively-charged PEI/TMC nanofiltration membrane prepared by adding a diamino-silane coupling agent for Li^+/Mg^{2+} separation. *J. Membr. Sci.* **672**, 121468 (2023).

84. P. Xu, J. Hong, X. Qian, Z. Xu, H. Xia, Q.-Q. Ni, "Bridge" graphene oxide modified positive charged nanofiltration thin membrane with high efficiency for Mg^{2+}/Li^+ separation. *Desalination* **488**, 114522 (2020).

85. P. Xu, J. Hong, Z. Xu, H. Xia, Q.-Q. Ni, Positively charged nanofiltration membrane based on (MWCNTs-COOK)-engineered substrate for fast and efficient lithium extraction. *Sep. Purif. Technol.* **270**, 118796 (2021).

86. S.-Y. Sun, L.-J. Cai, X.-Y. Nie, X. Song, J.-G. Yu, Separation of magnesium and lithium from brine using a Desal nanofiltration membrane. *J. Water Process Eng.* **7**, 210–217 (2015).

87. X. Li, C. Zhang, S. Zhang, J. Li, B. He, Z. Cui, Preparation and characterization of positively charged polyamide composite nanofiltration hollow fiber membrane for lithium and magnesium separation. *Desalination* **369**, 26–36 (2015).

88. B. K. Pramanik, M. B. Asif, S. Kentish, L. D. Nghiem, F. I. Hai, Lithium enrichment from a simulated salt lake brine using an integrated nanofiltration-membrane distillation process. *J. Environ. Chem. Eng.* **7**, 103395 (2019).

89. P. Xu, J. Hong, Z. Xu, H. Xia, Q.-Q. Ni, Novel aminated graphene quantum dots (GQDs-NH_2)-engineered nanofiltration membrane with high Mg^{2+}/Li^+ separation efficiency. *Sep. Purif. Technol.* **258**, 118042 (2021).

90. W. Huang, Z. Wang, F. Xie, H. Ding, W. Li, X. Liang, X. Ma, Z. Xu, High performance polyamide TFC reverse osmosis membrane fabricated on co-deposition hydrophilic modified polyethylene substrate. *Desalination* **538**, 115909 (2022).

91. C. Guo, N. Li, X. Qian, J. Shi, M. Jing, K. Teng, Z. Xu, Ultra-thin double Janus nanofiltration membrane for separation of Li^+ and Mg^{2+}: "Drag" effect from carboxyl-containing negative interlayer. *Sep. Purif. Technol.* **230**, 115567 (2020).

92. P. Xu, W. Wang, X. Qian, H. Wang, C. Guo, N. Li, Z. Xu, K. Teng, Z. Wang, Positive charged PEI-TMC composite nanofiltration membrane for separation of Li^{+} and Mg^{2+} from brine with high Mg^{2+}/Li^{+} ratio. *Desalination* **449**, 57–68 (2019).

93. H.-Z. Zhang, Z.-L. Xu, H. Ding, Y.-J. Tang, Positively charged capillary nanofiltration membrane with high rejection for Mg^{2+} and Ca^{2+} and good separation for Mg^{2+} and Li^{+}. *Desalination* **420**, 158–166 (2017).

94. H. Wu, Y. Lin, W. Feng, T. Liu, L. Wang, H. Yao, X. Wang, A novel nanofiltration membrane with [MimAP][Tf_2N] ionic liquid for utilization of lithium from brines with high Mg^{2+}/Li^{+} ratio. *J. Membr. Sci.* **603**, 117997 (2020).

95. W. Li, C. Shi, A. Zhou, X. He, Y. Sun, J. Zhang, A positively charged composite nanofiltration membrane modified by EDTA for $LiCl/MgCl_2$ separation. *Sep. Purif. Technol.* **186**, 233–242 (2017).

96. Q. Shen, S. Xu, Z. Xu, H. Zhang, Z. Dong, Novel thin-film nanocomposite membrane with water-soluble polyhydroxylated fullerene for the separation of Mg^{2+}/Li^{+} aqueous solution. *J. Appl. Polym. Sci.* **136**, 48029 (2019).

97. P. Xu, J. Hong, Z. Xu, H. Xia, Q.-Q. Ni, MWCNTs-COOK-assisted high positively charged composite membrane: Accelerating Li^{+} enrichment and Mg^{2+} removal. *Compos. Part B Eng.* **212**, 108686 (2021).

98. C. Guo, X. Qian, F. Tian, N. Li, W. Wang, Z. Xu, S. Zhang, Amino-rich carbon quantum dots ultrathin nanofiltration membranes by double "one-step" methods: Breaking through trade-off among separation, permeation and stability. *Chem. Eng. J.* **404**, 127144 (2021).

99. D. Lu, T. Ma, S. Lin, Z. Zhou, G. Li, Q. An, Z. Yao, Q. Sun, Z. Sun, L. Zhang, Constructing a selective blocked-nanolayer on nanofiltration membrane via surface-charge inversion for promoting Li^{+} permselectivity over Mg^{2+}. *J. Membr. Sci.* **635**, 119504 (2021).

100. F. Aghili, A. A. Ghoreyshi, B. Van Der Bruggen, A. Rahimpour, A highly permeable UiO-66-NH_2/polyethyleneimine thin-film nanocomposite membrane for recovery of valuable metal ions from brackish water. *Process Saf. Environ. Prot.* **151**, 244–256 (2021).

101. L. Wang, D. Rehman, P.-F. Sun, A. Deshmukh, L. Zhang, Q. Han, Z. Yang, Z. Wang, H.-D. Park, J. H. Lienhard, C. Y. Tang, Novel positively charged metal-coordinated nanofiltration membrane for lithium recovery. *ACS Appl. Mater. Interfaces* **13**, 16906–16915 (2021).

102. R. He, S. Xu, R. Wang, B. Bai, S. Lin, T. He, Polyelectrolyte-based nanofiltration membranes with exceptional performance in Mg^{2+}/Li^{+} separation in a wide range of solution conditions. *J. Membr. Sci.* **663**, 121027 (2022).

103. R. He, C. Dong, S. Xu, C. Liu, S. Zhao, T. He, Unprecedented Mg^{2+}/Li^{+} separation using layer-by-layer based nanofiltration hollow fiber membranes. *Desalination* **525**, 115492 (2022).

104. P. Cheng, T. Zhu, X. Wang, K. Fan, Y. Liu, X. Wang, S. Xia, Enhancing nanofiltration selectivity of metal–organic framework membranes via a confined interfacial polymerization strategy. *Environ. Sci. Technol.* **57**, 12879–12889 (2023).

105. B. Yuan, C. Jiang, P. Li, H. Sun, P. Li, T. Yuan, H. Sun, Q. J. Niu, Ultrathin polyamide membrane with decreased porosity designed for outstanding water-softening performance and superior antifouling properties. *ACS Appl. Mater. Interfaces* **10**, 43057–43067 (2018).

106. J. Zhu, J. Hou, S. Yuan, Y. Zhao, Y. Li, R. Zhang, M. Tian, J. Li, J. Wang, B. Van Der Bruggen, MOF-positioned polyamide membranes with a fishnet-like structure for elevated nanofiltration performance. *J. Mater. Chem. A* **7**, 16313–16322 (2019).

107. S. Gao, Y. Zhu, Y. Gong, Z. Wang, W. Fang, J. Jin, Ultrathin polyamide nanofiltration membrane fabricated on brush-painted single-walled carbon nanotube network support for ion sieving. *ACS Nano* **13**, 5278–5290 (2019).

108. S. Zhao, Z. Zhao, X. Zhang, Z. Zha, T. Tong, R. Wang, Z. Wang, Polyamide membranes with tunable surface charge induced by dipole–dipole interaction for selective ion separation. *Environ. Sci. Technol.* **58**, 5174–5185 (2024).

109. B. Yuan, S. Zhao, P. Hu, J. Cui, Q. J. Niu, Asymmetric polyamide nanofilms with highly ordered nanovoids for water purification. *Nat. Commun.* **11**, 6102 (2020).

110. J.-J. Wang, H.-C. Yang, M.-B. Wu, X. Zhang, Z.-K. Xu, Nanofiltration membranes with cellulose nanocrystals as an interlayer for unprecedented performance. *J. Mater. Chem. A* **5**, 16289–16295 (2017).

111. S. Wei, X. Ding, Y. Qiu, V. De Waele, H. Guo, Enhanced performance polyamide membrane by introducing high-porosity SOD/GO composite interlayer to tailor the interfacial polymerization process. *Chem. Eng. J.* **481**, 148595 (2024).

112. K. Fan, C. Pan, Y. Liu, X. Yuan, X. Wang, Y. Huang, P. Cheng, S. Xia, Exploration of a two-stage polymerization mechanism in construction of dense polyester membranes for drinking water treatment. *Desalination* **573**, 117203 (2024).

113. H.-Z. Zhang, Z.-L. Xu, Q. Shen, High-performance nanofiltration membrane intercalated by FeOOH nanorods for water nanofiltration. *Desalination* **498**, 114802 (2021).

114. K. Fan, Z. Mai, Y. Liu, X. Wang, Y. Huang, P. Cheng, S. Xia, H. Matsuyama, Fabrication of dense polyester nanofiltration membranes with superior fouling and chlorine resistance: Effect of polyol monomer properties and underlying mechanisms. *ACS EST Eng.* **3**, 1738–1747 (2023).

115. J. Yuan, M. Wu, H. Wu, Y. Liu, X. You, R. Zhang, Y. Su, H. Yang, J. Shen, Z. Jiang, Covalent organic framework-modulated interfacial polymerization for ultrathin desalination membranes. *J. Mater. Chem. A* **7**, 25641–25649 (2019).

116. Z. Sun, Q. Wu, C. Ye, W. Wang, L. Zheng, F. Dong, Z. Yi, L. Xue, C. Gao, Nanovoid membranes embedded with hollow zwitterionic nanocapsules for a superior desalination performance. *Nano Lett.* **19**, 2953–2959 (2019).

117. Z. Gu, P. Li, X. Gao, Y. Qin, Y. Pan, Y. Zhu, S. Yu, Q. Xia, Y. Liu, D. Zhao, G. Liu, Surface-crumpled thin-film nanocomposite membranes with elevated nanofiltration performance enabled by facilely synthesized covalent organic frameworks. *J. Membr. Sci.* **625**, 119144 (2021).

118. X. Wang, Q. Xiao, C. Wu, P. Li, S. Xia, Fabrication of nanofiltration membrane on MoS_2 modified PVDF substrate for excellent permeability, salt rejection, and structural stability. *Chem. Eng. J.* **416**, 129154 (2021).

119. D. Ren, X.-T. Bi, T.-Y. Liu, X. Wang, Oligo-ethylene-glycol based thin-film composite nanofiltration membranes for effective separation of mono-/di-valent anions. *J. Mater. Chem. A* **7**, 1849–1860 (2019).

120. S. Yang, Q. Jiang, K. Zhang, Few-layers 2D O–MoS_2 TFN nanofiltration membranes for future desalination. *J. Membr. Sci.* **604**, 118052 (2020).

121. Y. Zhu, W. Xie, S. Gao, F. Zhang, W. Zhang, Z. Liu, J. Jin, Single-walled carbon nanotube film supported nanofiltration membrane with a nearly 10 nm thick polyamide selective layer for high-flux and high-rejection desalination. *Small* **12**, 5034–5041 (2016).

122. J. Zhu, L. Qin, A. Uliana, J. Hou, J. Wang, Y. Zhang, X. Li, S. Yuan, J. Li, M. Tian, J. Lin, B. Van Der Bruggen, Elevated performance of thin film nanocomposite membranes enabled by modified hydrophilic MOFs for nanofiltration. *ACS Appl. Mater. Interfaces* **9**, 1975–1986 (2017).

123. M.-Q. Ma, C. Zhang, C.-Y. Zhu, S. Huang, J. Yang, Z.-K. Xu, Nanocomposite membranes embedded with functionalized MoS_2 nanosheets for enhanced interfacial compatibility and nanofiltration performance. *J. Membr. Sci.* **591**, 117316 (2019).

124. L. Zhang, M. Zhang, J. Lu, A. Tang, L. Zhu, Highly permeable thin-film nanocomposite membranes embedded with PDA/PEG nanocapsules as water transport channels. *J. Membr. Sci.* **586**, 115–121 (2019).

125. Q. Tang, X. An, H. Lan, H. Liu, J. Qu, A homogeneous carbon nitride nanomodifier for promoting the water permeation of polyamide desalination membranes. *Sep. Purif. Technol.* **343**, 127082 (2024).

126. Z.-M. Zhan, Z.-L. Xu, K.-K. Zhu, S.-M. Xue, C.-H. Ji, B.-Q. Huang, C. Y. Tang, Y.-J. Tang, Superior nanofiltration membranes with gradient cross-linked selective layer fabricated via controlled hydrolysis. *J. Membr. Sci.* **604**, 118067 (2020).

127. W. Fang, L. Shi, R. Wang, Mixed polyamide-based composite nanofiltration hollow fiber membranes with improved low-pressure water softening capability. *J. Membr. Sci.* **468**, 52–61 (2014).

128. X.-D. Weng, Y.-L. Ji, R. Ma, F.-Y. Zhao, Q.-F. An, C.-J. Gao, Superhydrophilic and antibacterial zwitterionic polyamide nanofiltration membranes for antibiotics separation. *J. Membr. Sci.* **510**, 122–130 (2016).

129. J. S. Trivedi, D. V. Bhalani, G. R. Bhadu, S. K. Jewrajka, Multifunctional amines enable the formation of polyamide nanofilm composite ultrafiltration and nanofiltration membranes with modulated charge and performance. *J. Mater. Chem. A* **6**, 20242–20253 (2018).

130. Z. Wang, Z. Wang, S. Lin, H. Jin, S. Gao, Y. Zhu, J. Jin, Nanoparticle-templated nanofiltration membranes for ultrahigh performance desalination. *Nat. Commun.* **9**, 2004 (2018).

131. J. Zhu, J. Hou, R. Zhang, S. Yuan, J. Li, M. Tian, P. Wang, Y. Zhang, A. Volodin, B. Van Der Bruggen, Rapid water transport through controllable, ultrathin polyamide nanofilms for high-performance nanofiltration. *J. Mater. Chem. A* **6**, 15701–15709 (2018).

132. K. Shen, C. Cheng, T. Zhang, X. Wang, High performance polyamide composite nanofiltration membranes via reverse interfacial polymerization with the synergistic interaction of gelatin interlayer and trimesoyl chloride. *J. Membr. Sci.* **588**, 117192 (2019).

133. L. Jia, X. Zhang, J. Zhu, S. Cong, J. Wang, J. Liu, Y. Zhang, Polyvinyl alcohol-assisted high-flux thin film nanocomposite membranes incorporated with halloysite nanotubes for nanofiltration. *Environ. Sci. Water Res. Technol.* **5**, 1412–1422 (2019).

134. C. Jiang, L. Tian, Z. Zhai, Y. Shen, W. Dong, M. He, Y. Hou, Q. J. Niu, Thin-film composite membranes with aqueous template-induced surface nanostructures for enhanced nanofiltration. *J. Membr. Sci.* **589**, 117244 (2019).

135. Z. Yang, Z. Zhou, H. Guo, Z. Yao, X. Ma, X. Song, S.-P. Feng, C. Y. Tang, Tannic acid/Fe^{3+} nanoscaffold for interfacial polymerization: Toward enhanced nanofiltration performance. *Environ. Sci. Technol.* **52**, 9341–9349 (2018).

136. X. Yang, Controllable interfacial polymerization for nanofiltration membrane performance improvement by the polyphenol interlayer. *ACS Omega* **4**, 13824–13833 (2019).

137. H. Peng, Q. Tang, S. Tang, J. Gong, Q. Zhao, Surface modified polyamide nanofiltration membranes with high permeability and stability. *J. Membr. Sci.* **592**, 117386 (2019).

138. S. Yuan, G. Zhang, J. Zhu, N. Mamrol, S. Liu, Z. Mai, P. Van Puyvelde, B. Van Der Bruggen, Hydrogel assisted interfacial polymerization for advanced nanofiltration membranes. *J. Mater. Chem. A* **8**, 3238–3245 (2020).

139. Y. Liu, J. Zhu, J. Zheng, X. Gao, J. Wang, X. Wang, Y. F. Xie, X. Huang, B. Van Der Bruggen, A facile and scalable fabrication procedure for thin-film composite membranes: integration of phase inversion and interfacial polymerization. *Environ. Sci. Technol.* **54**, 1946–1954 (2020).

140. B.-Q. Huang, Y.-J. Tang, Z.-X. Zeng, S.-M. Xue, S.-Q. Li, Y.-R. Wang, E.-C. Li, C. Y. Tang, Z.-L. Xu, Enhancing nanofiltration performance for antibiotics/NaCl separation via water activation before microwave heating. *J. Membr. Sci.* **629**, 119285 (2021).

141. Z. Zhang, X. Shi, R. Wang, A. Xiao, Y. Wang, Ultra-permeable polyamide membranes harvested by covalent organic framework nanofiber scaffolds: A two-in-one strategy. *Chem. Sci.* **10**, 9077–9083 (2019).

142. Y. Du, C. Zhang, Q. Zhong, X. Yang, J. Wu, Z. Xu, Ultrathin alginate coatings as selective layers for nanofiltration membranes with high performance. *ChemSusChem* **10**, 2788–2795 (2017).

143. K. Shen, P. Li, T. Zhang, X. Wang, Salt-tuned fabrication of novel polyamide composite nanofiltration membranes with three-dimensional turing structures for effective desalination. *J. Membr. Sci.* **607**, 118153 (2020).

144. X. Cao, J. Guo, J. Cai, M. Liu, S. Japip, W. Xing, S. Sun, The encouraging improvement of polyamide nanofiltration membrane by cucurbituril-based host–guest chemistry. *AIChE J.* **66**, e16879 (2020).

145. M. B. M. Y. Ang, C.-L. Tang, M. R. De Guzman, H. L. C. Maganto, A. R. Caparanga, S.-H. Huang, H.-A. Tsai, C.-C. Hu, K.-R. Lee, J.-Y. Lai, Improved performance of thin-film nanofiltration membranes fabricated with the intervention of surfactants having different structures for water treatment. *Desalination* **481**, 114352 (2020).

146. X. Yang, Monitoring the interfacial polymerization of piperazine and trimesoyl chloride with hydrophilic interlayer or macromolecular additive by in situ FT-IR spectroscopy. *Membranes* **10**, 12 (2020).

147. Y. Zhu, P. Dou, H. He, H. Lan, S. Xu, Y. Zhang, T. He, J. Niu, Improvement of permeability and rejection of an acid resistant polysulfonamide thin-film composite nanofiltration membrane by a sulfonated poly(ether ether ketone) interlayer. *Sep. Purif. Technol.* **239**, 116528 (2020).

148. Y. Zeng, L. Wang, L. Zhang, J. Q. Yu, An acid resistant nanofiltration membrane prepared from a precursor of poly(s-triazine-amine) by interfacial polymerization. *J. Membr. Sci.* **546**, 225–233 (2018).

149. X. Kong, M.-Y. Zhou, C.-E. Lin, J. Wang, B. Zhao, X.-Z. Wei, B.-K. Zhu, Polyamide/PVC based composite hollow fiber nanofiltration membranes: Effect of substrate on properties and performance. *J. Membr. Sci.* **505**, 231–240 (2016).

150. S. Zhu, S. Zhao, Z. Wang, X. Tian, M. Shi, J. Wang, S. Wang, Improved performance of polyamide thin-film composite nanofiltration membrane by using polyetersulfone/polyaniline membrane as the substrate. *J. Membr. Sci.* **493**, 263–274 (2015).

151. Z. Liao, X. Fang, J. Xie, Q. Li, D. Wang, X. Sun, L. Wang, J. Li, Hydrophilic hollow nanocube-functionalized thin film nanocomposite membrane with enhanced nanofiltration performance. *ACS Appl. Mater. Interfaces* **11**, 5344–5352 (2019).

152. Z. Yao, H. Guo, Z. Yang, W. Qing, C. Y. Tang, Preparation of nanocavity-contained thin film composite nanofiltration membranes with enhanced permeability and divalent to monovalent ion selectivity. *Desalination* **445**, 115–122 (2018).

153. C. Wang, Z. Li, J. Chen, Z. Li, Y. Yin, L. Cao, Y. Zhong, H. Wu, Covalent organic framework modified polyamide nanofiltration membrane with enhanced performance for desalination. *J. Membr. Sci.* **523**, 273–281 (2017).

154. J. Zhu, S. Yuan, A. Uliana, J. Hou, J. Li, X. Li, M. Tian, Y. Chen, A. Volodin, B. V. Der Bruggen, High-flux thin film composite membranes for nanofiltration mediated by a rapid co-deposition of polydopamine/piperazine. *J. Membr. Sci.* **554**, 97–108 (2018).

155. K. Shen, W. Hua, S. Ding, X. Wang, Customizing versatile polyamide nanofiltration membrane by the incorporation of a novel glycolic acid inhibitor. *Sep. Purif. Technol.* **255**, 117632 (2021).

156. M. Wu, T. Ma, Y. Su, H. Wu, X. You, Z. Jiang, R. Kasher, Fabrication of composite nanofiltration membrane by incorporating attapulgite nanorods during interfacial polymerization for high water flux and antifouling property. *J. Membr. Sci.* **544**, 79–87 (2017).

157. X. Cui, G. Kong, S. Wei, Z. Zhang, Z. Kang, H. Guo, PolyMOF interlayers modulated interfacial polymerization of ultra-thin nanofiltration membranes with efficient and stable desalination performance. *J. Membr. Sci.* **702**, 122780 (2024).

158. X.-G. Jin, X.-K. Liang, J.-H. Liu, J.-W. Mo, T.-X. Ren, X.-H. Ma, Z.-L. Xu, Development of high permeability nanofiltration membranes through porous 2D MOF nanosheets. *Chem. Eng. J.* **471**, 144566 (2023).

159. P. Sarkar, S. Modak, S. Karan, Ultraselective and highly permeable polyamide nanofilms for ionic and molecular nanofiltration. *Adv. Funct. Mater.* **31**, 2007054 (2021).

160. F. Peng, X. Huang, A. Jawor, E. M. V. Hoek, Transport, structural, and interfacial properties of poly(vinyl alcohol)–polysulfone composite nanofiltration membranes. *J. Membr. Sci.* **353**, 169–176 (2010).

161. S. Jeon, C. H. Park, S.-H. Park, M. G. Shin, H.-J. Kim, K.-Y. Baek, E. P. Chan, J. Bang, J.-H. Lee, Star polymer-assembled thin film composite membranes with high separation performance and low fouling. *J. Membr. Sci.* **555**, 369–378 (2018).

162. Y. Li, E. Wong, Z. Mai, B. Van Der Bruggen, Fabrication of composite polyamide/Kevlar aramid nanofiber nanofiltration membranes with high permselectivity in water desalination. *J. Membr. Sci.* **592**, 117396 (2019).

163. C. Boo, Y. Wang, I. Zucker, Y. Choo, C. O. Osuji, M. Elimelech, High performance nanofiltration membrane for effective removal of perfluoroalkyl substances at high water recovery. *Environ. Sci. Technol.* **52**, 7279–7288 (2018).

164. X. Kong, Z.-L. Qiu, C.-E. Lin, Y.-Z. Song, B.-K. Zhu, L.-P. Zhu, X.-Z. Wei, High permselectivity hyperbranched polyester/polyamide ultrathin films with nanoscale heterogeneity. *J. Mater. Chem. A* **5**, 7876–7884 (2017).

165. B. Yuan, S. Zhang, C. Jiang, P. Hu, J. Cui, S. Zhao, N. Wang, Q. J. Niu, Alicyclic polyamide nanofilms with an asymmetric structure for Cl^-/SO_4^{2-} separation. *AIChE J.* **68**, e17419 (2022).

166. L. Zhang, R. Zhang, M. Ji, Y. Lu, Y. Zhu, J. Jin, Polyamide nanofiltration membrane with high mono/divalent salt selectivity via pre-diffusion interfacial polymerization. *J. Membr. Sci.* **636**, 119478 (2021).

167. P. Xu, S. Duan, Z. Li, M. Hu, P. Zhang, L. Dai, Z. Mai, K. Guan, H. Matsuyama, Charge-sign-independent separation of mono- and divalent ions with nanofiltration membranes. *Adv. Funct. Mater.* **35**, 2416458 (2025).

168. X. You, K. Xiao, H. Wu, Y. Li, R. Li, J. Yuan, R. Zhang, Z. Zhang, X. Liang, J. Shen, Z. Jiang, Electrostatic-modulated interfacial polymerization toward ultra-permselective nanofiltration membranes. *iScience* **24**, 102369 (2021).

169. Y. Pan, R. Xu, Z. Lü, S. Yu, M. Liu, C. Gao, Enhanced both perm-selectivity and fouling resistance of poly(piperazine-amide) nanofiltration membrane by incorporating sericin as a co-reactant of aqueous phase. *J. Membr. Sci.* **523**, 282–290 (2017).

170. Y. Du, Y. Lv, W.-Z. Qiu, J. Wu, Z.-K. Xu, Nanofiltration membranes with narrowed pore size distribution via pore wall modification. *Chem. Commun.* **52**, 8589–8592 (2016).

171. Z.-L. Qiu, W.-H. Yu, Y.-J. Shen, B.-K. Zhu, L.-F. Fang, Janus charged polyamide nanofilm with ultra-high separation selectivity for mono−/divalent ions. *Chem. Eng. J.* **416**, 129023 (2021).

172. S. Xu, H. Lin, G. Li, J. Wang, Q. Han, F. Liu, Anionic covalent organic framework as an interlayer to fabricate negatively charged polyamide composite nanofiltration membrane featuring ions sieving. *Chem. Eng. J.* **427**, 132009 (2022).

173. G. Gong, P. Wang, Z. Zhou, Y. Hu, New insights into the role of an interlayer for the fabrication of highly selective and permeable thin-film composite nanofiltration membrane. *ACS Appl. Mater. Interfaces* **11**, 7349–7356 (2019).

174. S. Liang, G. Xu, Y. Jin, Z. Wu, Z. Cai, N. Zhao, Z. Wu, Annealing of supporting layer to develop nanofiltration membrane with high thermal stability and ion selectivity. *J. Membr. Sci.* **476**, 475–482 (2015).

175. Y.-X. Fang, Z.-L. Xu, S.-J. Xu, Polyamide nanofiltration membrane modified with defective covalent organic framework interlayer for selective ion sieving. *Sep. Purif. Technol.* **355**, 129747 (2025).

176. L. Li, G. Zhu, Y. Tong, K. Ding, Z. Wang, C. Meng, C. Gao, Polyethyleneimine modified polyamide composite nanofiltration membrane for separation of lithium and magnesium. *J. Water Process Eng.* **54**, 103894 (2023).

177. Y. Li, M. Wang, X. Xiang, Y. J. Zhao, Z. J. Peng, Separation performance and fouling analyses of nanofiltration membrane for lithium extraction from salt lake brine. *J. Water Process Eng.* **54**, 104009 (2023).

178. G. Bargeman, J. M. Vollenbroek, J. Straatsma, C. G. P. H. Schroën, R. M. Boom, Nanofiltration of multi-component feeds. Interactions between neutral and charged components and their effect on retention. *J. Membr. Sci.* **247**, 11–20 (2005).

179. X. Wen, P. Ma, C. Zhu, Q. He, X. Deng, Preliminary study on recovering lithium chloride from lithium-containing waters by nanofiltration. *Sep. Purif. Technol.* **49**, 230–236 (2006).

180. M. Awais Ashraf, M. Usman, I. Hussain, F. Ahmad, S. Guo, L. Zhang, Lithium extraction from high magnesium salt lake brine with an integrated membrane technology. *Sep. Purif. Technol.* **302**, 122163 (2022).

181. Y. Zhao, H. Wang, Y. Li, M. Wang, X. Xiang, An integrated membrane process for preparation of lithium hydroxide from high Mg/Li ratio salt lake brine. *Desalination* **493**, 114620 (2020).
